# Strong Bonding of Lattice N Activates Metal Ni to Achieve Efficient Water Splitting

**DOI:** 10.1002/advs.202411526

**Published:** 2024-11-22

**Authors:** Niandan Zhao, Wei Luo, Sijun Li, Hua Wang, Yini Mao, Yimin Jiang, Wenbin Wang, Ming Li, Wei Su, Rongxing He

**Affiliations:** ^1^ Key Laboratory of Luminescence Analysis and Molecular Sensing (Southwest University), Ministry of Education, College of Chemistry and Chemical Engineering Southwest University Chongqing 400715 China; ^2^ Key Laboratory of Beibu Gulf Environment Change and Resources Utilization (Nanning Normal University), Ministry of Education, College of Chemistry and Life Science Nanning Normal University 175 Mingxiu East Road Nanning 530000 China

**Keywords:** lattice N, Ni activation, N─Ni©NC, overall water splitting, strong bonding

## Abstract

Developing efficient and robust free‐standing electrocatalysts for overall water splitting is a promising but challenging task. Herein, the N‐incorporated Ni nanosheets non‐fully encapsulated by N‐doped carbon (NC) layer are fabricated (N─Ni©NC). The introduction of N not only regulates the size of nanosheets in N─Ni©NC but also promotes the electrochemical activity of metal Ni. Experimental and theoretical results reveal that strong bonding of the lattice N activates the inert metal Ni by promoting charge transfer between Ni and N. In addition, the upward shift of the d‐band center induced by lattice N enhances the adsorption of intermediates, thereby making Ni as a new OER active site together with C. This strategy of generating Ni and C dual active sites by introducing lattice N greatly accelerates oxygen evolution reaction (OER) kinetics, resulting in excellent electrocatalytic performance of N─Ni©NC. At the current density of 10 mA cm^−2^, the overpotentials of hydrogen evolution reaction (HER) and OER are 27 and 206 mV, respectively, and the cell voltage for overall water splitting only needs 1.47 V. This work offers a unique heteroatom activation approach for designing free‐standing electrodes with high activity.

## Graphical abstract

A free‐standing electrode N─Ni©NC is constructed, which displays excellent hydrogen evolution reaction (HER) and oxygen evolution reaction (OER) performance. The strong bonding of the lattice N activates the inert metal Ni by promoting charge transfer between Ni and N, thereby making Ni as a new OER active site together with C. This strategy of generating dual active sites by introducing lattice N greatly accelerates OER kinetics.

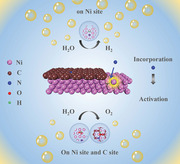

## 1 Introduction

Producing green hydrogen without carbon emission via water electrolysis is a viable method.^[^ ^1^, ^2^, ^3^, ^4^ ^]^ For the purpose of enhancing water electrolysis efficiency, it is crucial to develop a bifunctional electrocatalyst with excellent hydrogen evolution reaction (HER) and oxygen evolution reaction (OER) performance.^[^ ^5^, ^6^, ^7^, ^8^ ^]^ Ni‐based materials, as one of the candidates, have great potential in HER. Such as metallic nickel,^[^ ^9^, ^10^ ^]^ nickel phosphide,^[^ ^11^, ^12^ ^]^ nickel sulfide.^[^ ^13^, ^14^, ^15^ ^]^ and nickel nitride.^[^ ^16^, ^17^ ^]^ Nevertheless, the OER property of Ni‐based catalysts, especially the metal Ni, is usually inferior, making them difficult to serve as bifunctional electrocatalysts. Although some Ni‐based materials with excellent OER performance have been reported, the improvement in their activity mostly comes from the introduction of new components, rather than enhancing the inherent activity of Ni. Therefore, the key for designing Ni‐based bifunctional electrocatalysts is to simultaneously boost the HER and OER performance, especially to activate the OER activity.

As is well known, the presence of high valence Ni is conducive to promoting the OER performance of Ni‐based catalysts.^[^ ^18^, ^19^ ^]^ Therefore, finding a suitable strategy to obtain a high valence state of Ni can essentially activate the metal Ni. Doping non‐metallic elements like S, N, and P can regulate the electronic structure of catalysts and improve their intrinsic activity, which has become an attractive method.^[^ ^20^, ^21^, ^22^ ^]^ At present, great progress has been made in enhancing the electrocatalytic activity of materials by using the N doping strategy. For instance, Yang et al. prepared N‐doped Co_3_O_4_ electrocatalyst enriched with defects. N‐doping adjusts the electronic state of Co atoms, generating a large number of oxygen vacancies that activate lattice oxygen, thereby accelerating reaction kinetics.^[^ ^23^ ^]^ Liu et al. doped trace N into NiCoP and synthesized bifunctional catalysts. The addition of N regulates electron density of Ni, and P and facilitates the desorption of HER intermediates. Additionally, trace N doping reduces the thermodynamic barrier of proton‐coupled electron transfer and promotes O─O bond formation, thus effectively improving the OER performance.^[^ ^24^ ^]^ Consequently, introducing N with strong electron withdrawing ability into the cayalyst is expected to make high valence Ni a new OER active site through Ni‐N interaction. Although the incorporation of N atoms boosts the electrocatalytic activity of Ni‐based materials, there are still some issues to be addressed urgently to meet the practical application. First, metal Ni nanoparticles are prone to agglomeration, hindering active site exposure. Furthermore, the Ni‐based catalysts remain weak in terms of durability under the extreme catalytic conditions of alkaline solutions. Therefore, it is vital to construct free‐standing electrodes that can uniformly disperse the active substances and cover them with a carbon layer to enhance the stability of the core material.

Various methods have been adopted to develop free‐standing electrocatalysts, including the use of biomass cellulose.^[^ ^25^, ^26^, ^27^ ^]^ Biomass cellulose contains substantial functional groups that can provide growth sites for oxides and hydroxides.^[^ ^28^, ^29^ ^]^ The formation of cellulose/metal hydroxide through chemical bonds not only inhibits nanoparticles from aggregating but also avoids the use of binders.^[^ ^30^, ^31^ ^]^ Cellulose, like glucose, dicyandiamide, and melamine, forms a carbon protective layer after high‐temperature pyrolysis, improving the stability of the core material.^[^ ^32^, ^33^, ^34^, ^35^ ^]^ Adding other N source when calcining the cellulose/metal hydroxide, the free‐standing electrode of N‐incorporated metal coated by N‐doped carbon (NC) layer can be obtained. The introduction of N may enter the metal lattice, which has the opportunity to radically boost the electrocatalytic activity of metal. In addition, the generated NC layer can improve the wettability and stability of the material.

In this work, N‐incorporated Ni nanosheets that are non‐fully wrapped by NC layer (N─Ni©NC) were prepared by nitrogenization of cellulose/Ni(OH)_2_(Cel/Ni(OH)_2_). Impressively, N─Ni©NC was used directly as an electrode presents low overpotentials (27 mV for HER and 206 mV for OER at 10 mA cm^−2^ current density) and superb stability. The density functional theory (DFT) calculations reveal that the strong bonding between lattice N and Ni triggers a redistribution of charges, leading to the formation of high valence Ni, which facilitates the switch of Ni site to a highly efficient active site for OER. Moreover, the lattice N also retains the C active site, which together with the activated Ni serve as OER active centers, greatly enhancing the OER property of N─Ni©NC.

## 2 Results and Discussion

### 2.1 Synthesis and Characterization

**Figure 1:**
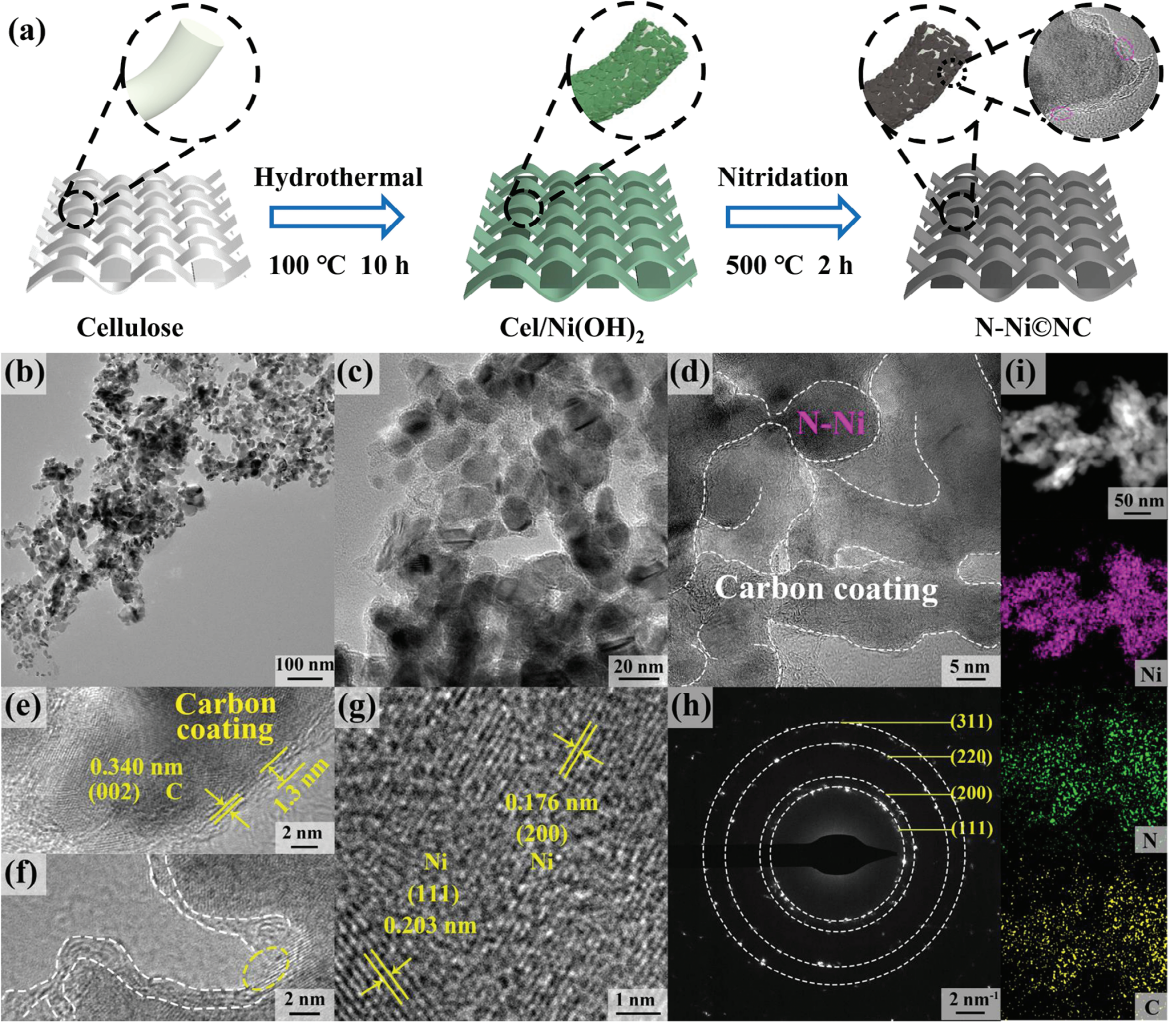
a) Schematic diagram for the synthesis of N─Ni©NC. b–g) TEM and HRTEM images, h) SAED pattern, and i) HAADF‐STEM image and EDS‐mapping pictures of N─Ni©NC.

In **Figure** **1**a, the N─Ni©NC preparation process was presented. The Cel/Ni(OH)_2_ precursor was synthesized from biomass cellulose and Ni sources by hydrothermal. The cellulose surface features an even distribution of the in situ grown Ni(OH)_2_ nanosheets (Figure S1, Supporting Information). Then, the N─Ni©NC catalyst was obtained by nitriding at 500 °C for 2 h.

The morphology of the catalysts was investigated by sacnning electron microscope (SEM) and transmission electron microscopy (TEM). N─Ni nanosheets grown without cellulose show obvious agglomeration (Figure S2, Supporting Information), while the nanosheets in N─Ni©NC are evenly distributed (Figure S3, Supporting Information), suggesting that using cellulose as the substrate can prevent the aggregation of nanosheets. The role of cellulose in the preparation of N─Ni©NC free‐standing electrocatalysts is explained in the supporting information (Figures S4, S5, Supporting Information). To figure out the impact of the N incorporation on the morphology of the catalyst, the amount of urea used in the calcination was changed to obtain N─Ni©NC‐n (Figure S6, Supporting Information). The size of the nanosheets in Ni©C is ≈10 times that in N─Ni©NC. As a consequence, the presence of N adjusts the size of the nanosheets in the catalyst, thus exposing more accessible sites and increasing the area in contact with the electrolyte. The Brunauer‐Emmett‐Teller (BET) specific surface area of N─Ni©NC and Ni©C are 31.9 and 4.0 m^2^ g^−1^, respectively (Figure S7, Supporting Information), testifying that the specific surface area of catalyst can increase with the addition of N, which is helpful to improve the electrocatalytic performance. The morphology of N─Ni©NC presented in Figure 1b is consistent with that observed in SEM. Figure 1c,d clearly show the nanosheets and the coating layer (marked by white lines). The lattice fringes of the coating layer are measured to be 0.340 nm (Figure 1e), which is attributed to the (002) plane of C material.^[^ ^36^ ^]^ Note that the nanosheet is non‐fully encapsulated by carbon layer, where the white dotted lines denote the carbon layer and the yellow circles represent the bare nanosheet (Figure 1f; Figure S8, Supporting Information). During the synthesis process, the feed amount of cellulose was halved to obtain N─Ni©NC‐0.5C with a lower wrapping degree of carbon layer than N─Ni©NC (Figure S9, Supporting Information). The high‐resolutoin TEM (HRTEM) of Figure 1g confirms that the interplanar spacing of 0.176 and 0.203 nm is assigned to the (200) and (111) planes of Ni.^[^ ^9^ ^]^ In the selected area electron diffraction (SAED) pattern of Figure 1h, there are distinct diffraction rings belonging to the (111), (200), (220), and (311) planes of Ni, respectively. The energy dispersive X‐ray spectra (EDS)‐mapping pictures prove that Ni, C and N elements exist in the nanosheets (Figure 1i), which is in accordance with the X‐ray photoelectron spectroscopy (XPS) analysis.

**Figure 2:**
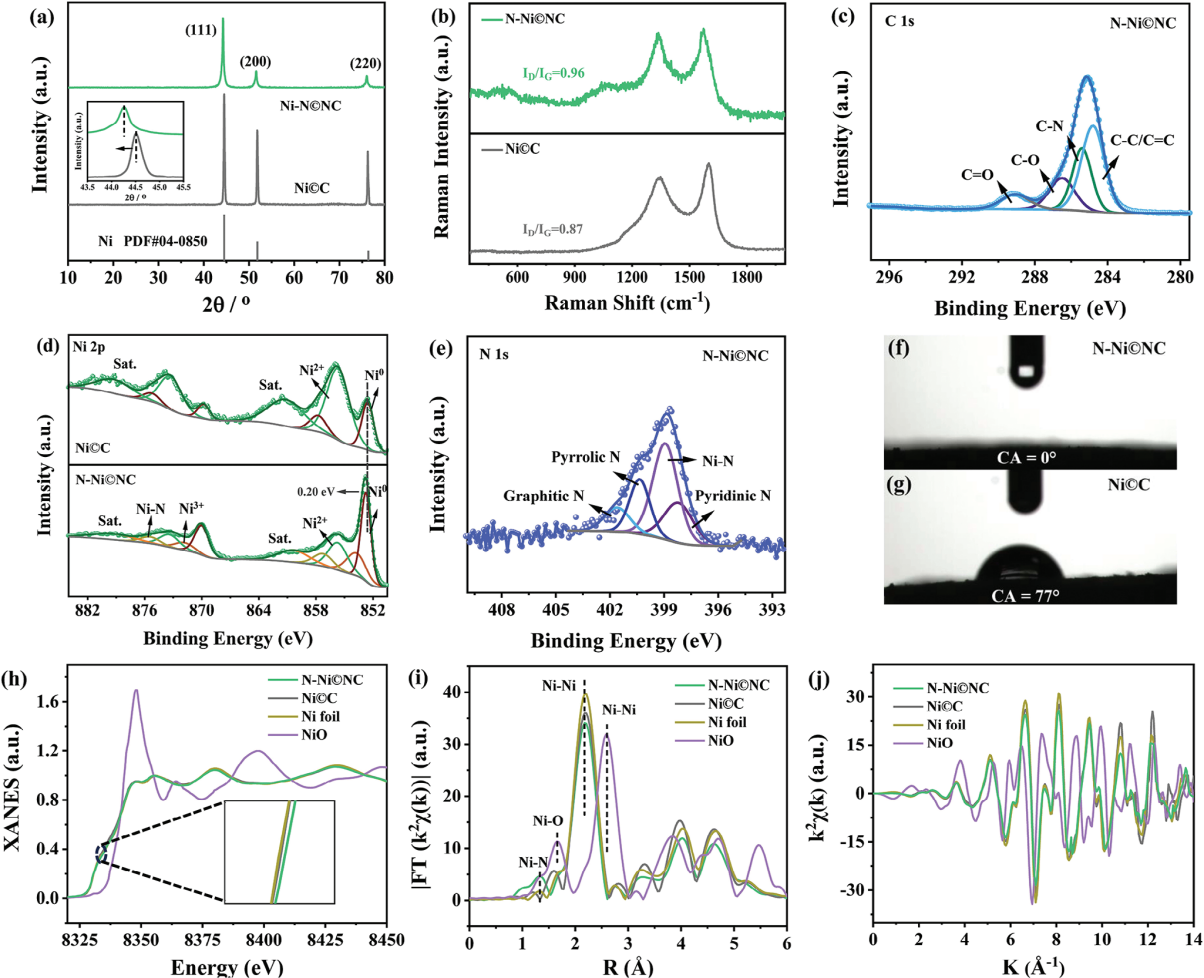
a) XRD patterns, b) Raman and XPS spectra of N─Ni©NC and Ni©C, c) C 1s, d) Ni 2p, and e) N 1s. Contact angle images of f) N─Ni©NC and g) Ni©C. h) Ni K‐edge XANES spectra for N─Ni©NC, Ni©C, Ni foil, and NiO, the rising edge of the XANES spectra is magnified in the inset image. i) R space and corresponding inverse FT‐EXAFS results of N─Ni©NC, Ni©C, Ni foil, and NiO. j) Ni K‐edge EXAFS oscillations spectra for N─Ni©NC, Ni©C, Ni foil, and NiO.

The structural information of all samples was determined by X‐ray diffraction (XRD). In **Figure** **2**a, the peaks of Ni©C located at 2θ = 44.5°, 51.8° and 76.3° correspond to the (111), (200) and (220) planes of Ni (PDF#04‐0850). Compared with Ni©C, the diffraction peaks of N─Ni©NC shift toward a lower angle (as shown in the illustration), indicating the introduction of N causes the lattice expansion of Ni crystal (Figure S10, Supporting Information).^[^ ^37^ ^]^ Considering that N has a smaller atomic radius than Ni, it is most likely that the N atom enters the lattice center of Ni crystal instead of replacing Ni atom. Both N─Ni©NC and Ni©C display the typical D and G carbon bands in the Raman spectra (Figure 2b), and the addition of N increases I_D_/I_G_ value from 0.87 to 0.96, which is associated with the more defects formed after N doping into C layer.

The structure of the catalyst was further analyzed by the XPS characterization to learn more about the influence of N atom incorporation. Only the peaks of C 1s, O 1s and Ni 2p emerge in the XPS survey of Ni©C (Figure S11a, Supporting Information), but an additional peak of N 1s appears in N─Ni©NC, manifesting that these elements are contained in the counterpart material. The peaks located at 284.8, 285.4, 286.5, and 289.1 eV in the high‐resolution C 1s spectrum of N─Ni©NC (Figure 2c) are ascribed to C─C/C═C, C─N, C─O, and C═O,^[^ ^38^ ^]^ which once again confirms that N enters the C layer, consistent with the Raman results. In Figure 2d, the Ni 2p spectrum in Ni©C can be fitted into multiple peaks related to Ni^0^ (852.5 and 869.8 eV), Ni^2+^ (855.7 and 873.3 eV) and their satellite peaks. For N─Ni©NC, the peaks attributed to Ni^0^ (852.7 and 870.0 eV) move to the higher binding energy with respect to their counterparts in Ni©C, verifying that lattice N causes a redistribution of electron density at the Ni sites, which is likely to activate the inert Ni. Furthermore, the peaks located at 853.8 and 871.8 eV manifest the existence of Ni─N bond, which provides an efficient basis for charge transfer between Ni and N. The peaks at 857.2 and 875.4 eV belong to Ni^3+^. The presence of Ni^3+^ indicates that the addition of N atoms synchronously triggers the generation of Ni vacancies,^[^ ^39^, ^40^ ^]^ which is further supported by the electron paramagnetic resonance (EPR) spectrum (Figure S12, Supporting Information). Evidently, our previous speculation that N enters the Ni lattice of the N─Ni©NC catalyst is verified. According to literature reports, lattice N may indeed cause the absence of Ni atoms, resulting in the Ni vacancy.^[^ ^41^ ^]^ Further, no N signal was observed in the high‐resolution N 1s spectrum of Ni©C (Figure S11c, Supporting Information), while the peak at 398.9 eV assigned to Ni─N bond appears in the N 1s spectrum of N─Ni©NC (Figure 2e), indicating that N has been successfully introduced into the catalyst. In addition, three peaks at 398.2, 400.4, and 401.6 eV are corresponding to pyridinic N, pyrrolic N, and graphitic N, respectively. Pyridine N has excellent wettability,^[^ ^42^ ^]^ it can generate a large contact area with electrolyte, which speeds up the interface electron transfer between catalyst and electrolyte during the catalytic process, thereby improving catalytic activity. The enhanced surface wettability of N─Ni©NC can be corroborated by the contact angle (CA) testing. In Figure 2f, CA is close to 0° for N─Ni©NC, whereas a water droplet observed on the Ni©C surface shows a larger CA of 77° (Figure 2g), confirming that the former has a superhydrophilic property due to the addition of N in C layer. The superb wettability of N─Ni©NC helps to adsorb electrolyte on the catalyst surface, thus allowing more active sites to contact electrolyte and accelerating the overall water splitting.

X‐ray absorption near‐edge structure (XANES) was carried out to investigate the intrinsic structure of the catalyst at the atomic level. In Figure 2h, the absorption threshold position of Ni©C at the Ni *K*‐edge is almost the same as that of Ni foil, indicating that the oxidation state of Ni in Ni©C hardly changes, and Ni^0^ is the main valence state. However, the position of the absorption threshold of N─Ni©NC moves toward higher energy, demonstrating the slightly increased valence of Ni due to the transfer of electrons to the adjacent N atoms.^[^ ^43^, ^44^ ^]^ The existence of high‐valence Ni makes it possible to be efficient active sites in electrocatalytic reactions. In the Fourier transform of extended X‐ray absorption fine structure (FT‐EXAFS) spectrum (Figure 2i), there is no characteristic peak of Ni‐N in Ni©C. Nevertheless, for N─Ni©NC, in addition to the dominant Ni‐Ni peak at near 2.18 Å, another peak located at 1.35 Å comes from the Ni─N coordination (the fitting bond length is 1.81 Å), which confirms the strong bonding of lattice N with Ni. Considering that N has a stronger electronegativity than Ni, it can be inferred that electrons will transfer from Ni to N, resulting in a high valence Ni to promote OER. The oscillating frequencies and shapes of the *k* ^2^χ(*k*) oscillation curves at the Ni *K*‐edge of N─Ni©NC are different from those of Ni foil and NiO (Figure 2j), manifesting that the incorporating of N changes the coordination environment of Ni and may enhance the intrinsic activity of Ni. The EXAFS fitting results were analyzed to further illustrate the emergence of Ni vacancy (Table S1, Supporting Information). The coordination number of Ni sites decreases from 9.1 of Ni©C to 8.2 of N─Ni©NC due to the lattice N causes the formation of Ni vacancies.^[^ ^23^ ^]^ The above results uncover that the lattice N not only regulates the local electronic environment but also leads to the generation of Ni vacancy, which is crucial for the improvement of electrocatalytic performance. Additionally, these findings offer a reference for the construction of subsequent DFT calculation models.

### 2.2 Electrochemical Performance

**Figure 3:**
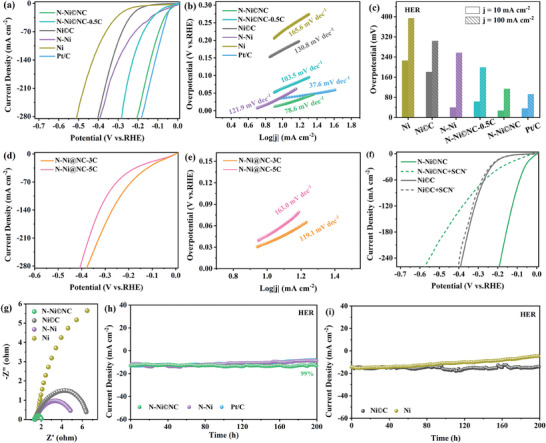
a) HER polarization curves of N─Ni©NC, N─Ni©NC‐0.5C, Ni©C, N─Ni, Ni, and Pt/C. b) Tafel plots. c) Overpotential histogram at the current density of 10 and 100 mA cm^−2^. d) HER polarization curves of N─Ni@NC‐3C and N─Ni@NC‐5C. e) Tafel plots. f) HER polarization curves of N─Ni©NC+SCN^−^ and Ni©C+SCN^−^. g) Nyquist plots of N─Ni©NC, Ni©C, N─Ni, and Ni at an overpotential of 200 mV. h) *I*‐*t* curves for N─Ni©NC, N─Ni, and Pt/C. i) *I*‐*t* curves for Ni©C and Ni.

The electrocatalytic HER performance of N─Ni©NC was first assessed. The optimization of N incorporation concentration indicates that the optimal catalytic property is attained when the urea dosage is 6.0 g (Figure S13, Supporting Information), therefore, N─Ni©NC is used for subsequent electrochemical tests. To reveal the effect of non‐fully encapsulated NC layer on catalyst performance, N─Ni@NC‐3C and N─Ni@NC‐5C fully coated with NC layer were prepared by increasing the carbon sources three and five times (Figures S14, S15, Supporting Information), and their performance was tested. In **Figure** **3**a, N─Ni©NC only requires an overpotential of 27 and 114 mV at the current density of 10 and 100 mA cm^−2^ (Figure 3c), respectively, which is comparable to the benchmark Pt/C (36 and 92 mV) and surpasses N─Ni©NC‐0.5C, Ni©C, Ni, N─Ni and the majority of currently reported Ni‐based catalysts (Table S2, Supporting Information). The above outcomes prove that the introduction of N can boost the HER activity. Compared with N─Ni©NC, the overpotential of N─Ni@NC‐3C at the current densities of 10 and 100 mA cm^−2^ increased by 9 and 115 mV (Figure 3d), respectively. Further, N─Ni@NC‐5C with fully wrapped thicker NC layers has worse HER activity. This indicates that the introduction of N is likely to make Ni become the HER active sites, while the fully wrapped NC layer will hinder the exposure of the Ni active sites, thereby weakening HER activity. It is well known that SCN^−^ can combine with the Ni center to form a complex, preventing the metal site from participating in the reaction, resulting in reduced electrochemical activity. Therefore, we conducted the SCN^−^ poisoning experiments, and the results (see Figure 3f) show that the HER performance of Ni©C+SCN^−^ is almost unaffected, while that of N─Ni©NC+SCN^−^ is significantly reduced, proving that Ni is the active center of N─Ni©NC. It was observed that N─Ni©NC has a smaller Tafel slope of 78.6 mV dec^−1^, which is somewhat higher than Pt/C but superior to N─Ni©NC‐0.5C, Ni©C, N─Ni, Ni, N─Ni@NC‐3C, and N─Ni@NC‐5C (Figure 3b,e), demonstrating its fast reaction kinetics. Furthermore, the electrochemical impedance spectroscopy (EIS) analysis shows that N─Ni©NC possesses the smallest charge transfer resistance of all the materials (Figure 3g), confirming that the incorporation of N and the coating of NC layer accelerate electron transfer. As a vital parameter to evaluate catalyst performance, the chronoamperometry tests were performed. The current density of N─Ni and Ni decays sharply, but after 200 h of continuous electrolysis, the decrease in current density of Ni©C and N─Ni©NC is negligible, indicating the excellent durability, which originates from the protection of the non‐fully encapsulated NC layer (Figure 3h,i). Moreover, N─Ni©NC also displays superb long‐term stability at higher voltages (Figure S16, Supporting Information). In brief, the unique structure of N─Ni©NC makes it have outstanding electrochemical activity and excellent stability.

**Figure 4:**
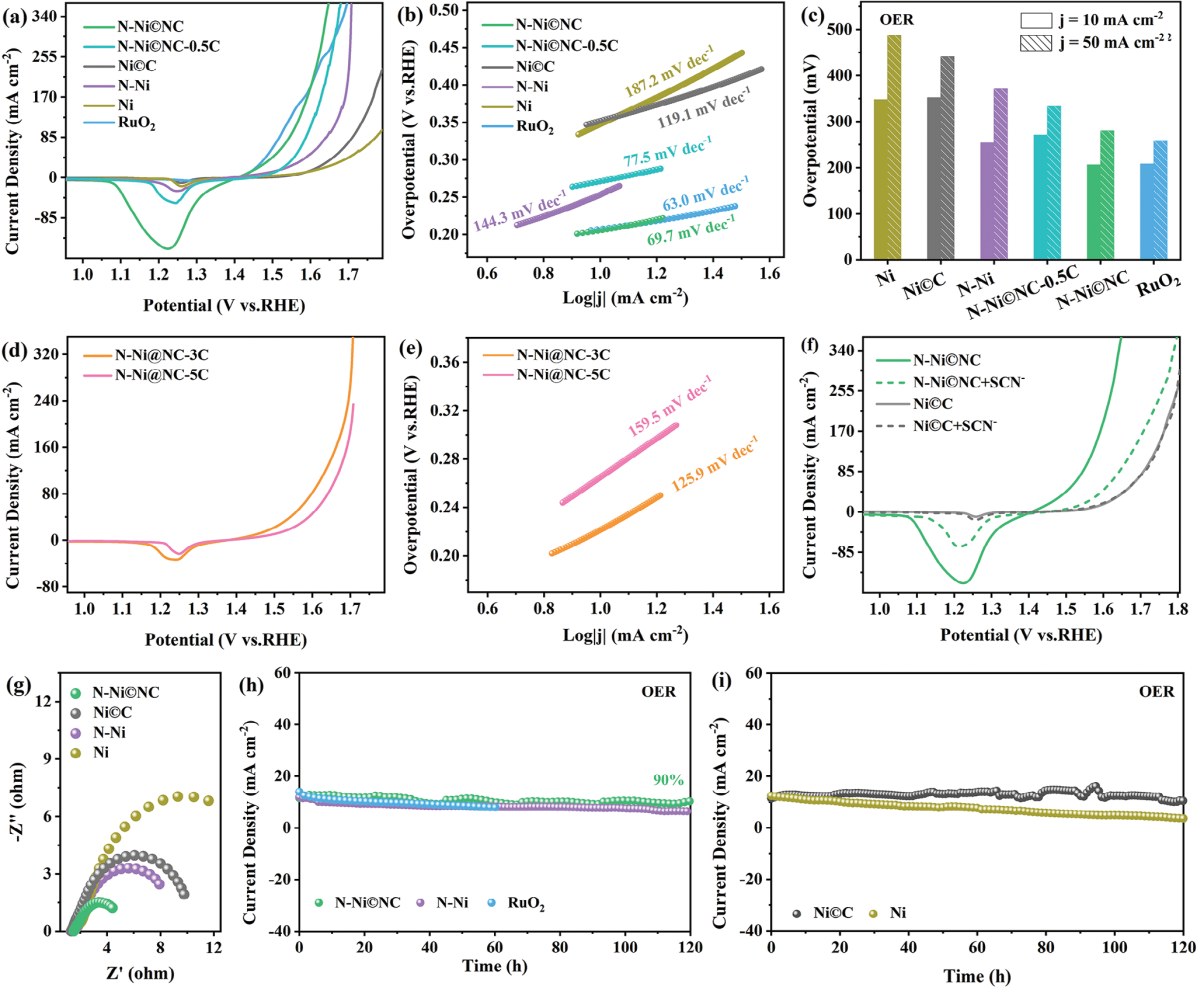
a) OER polarization curves of N─Ni©NC‐0.5C, N─Ni©NC, Ni©C, N─Ni, Ni and RuO_2_. b) Tafel plots. c) Overpotential histogram at the current density of 10 and 50 mA cm^−2^. d) OER polarization curves of N─Ni@NC‐3C and N─Ni@NC‐5C. e) Tafel plots. f) OER polarization curves of N─Ni©NC+SCN^−^ and Ni©C+SCN^−^. g) Nyquist plots of N─Ni©NC, Ni©C, N—Ni, and Ni at an overpotential of 300 mV. h) *I*‐*t* curves for N─Ni©NC, N—Ni, and RuO_2_. i) *I*‐*t* curves for Ni©C and Ni.

The OER activity of the catalyst was also evaluated, taking RuO_2_ as the benchmark. N─Ni©NC shows the overpotential of 206 and 280 mV at the current densities of 10 and 50 mA cm^−2^ (**Figure** **4**a; Figure S17, Supporting Information), which is similar to that of RuO_2_ (208 and 259 mV), much lower than N─Ni©NC‐0.5C, Ni©C and Ni, and superior to N─Ni (Figure 4c). These results manifest that the high valence Ni generated by the strong bonding between lattice N and Ni may induce it to become the active site, thereby significantly improving the OER performance of N─Ni©NC. Not only that, the OER property of N─Ni©NC also ranks among the top in numerous Ni‐based catalysts (Table S3, Supporting Information). The overpotential of N─Ni@NC‐3C (222 and 329 mV) only increases slightly after fully wrapping of thin NC layer, whereas the OER performance of N─Ni@NC‐5C is inferior owing to the obstruction of Ni active sites by the fully covered thicker NC layer (Figure 4d). Importantly, at the current density of 10 mA cm^−2^, the OER overpotential of N─Ni@NC‐3C increases by 7.2% in comparison to that of N─Ni©NC, which is much smaller than the increase in its HER overpotential compared with that of N─Ni©NC (33.3%). This result means that the NC layer is essential to enhancing the OER activity of N─Ni©NC,^[^ ^43^ ^]^ possibly because in addition to Ni, it is also the OER active center. In order to further clarify the active site of the catalyst in OER, the SCN^−^ poisoning experiments were performed again (Figure 4f). After SCN^−^ treatment, the change in the OER performance of Ni©C can be ignored, which is probably because its active site is C, while the OER performance of N─Ni©NC significantly decreased, indicating that Ni is indeed activated by lattice N and acts as a new OER active center. Although the Ni site is inactivated due to poisoning, the OER activity of N─Ni©NC+SCN^−^ is close to that of Ni©C, indicating that C is also an active site of OER. In short, there are Ni and C dual active sites in N─Ni©NC. Figure 4b,e shows that although the Tafel slope of N─Ni©NC (69.7 mV dec^−1^) is a little higher than that of RuO_2_, it is markedly lower than those of N─Ni©NC‐0.5C, Ni©C, Ni, N─Ni, N─Ni@NC‐3C, and N─Ni@NC‐5C, demonstrating that the reaction kinetics can be boosted by introducing N and partially encapsulated NC layers. In addition, N─Ni©NC has the smallest charge transfer resistance compared with those of Ni©C, Ni, and N─Ni, indicating better OER kinetics, which is in agreement with the results of the Tafel slope (Figure 4g). The stability of Ni and N─Ni (Figure 4h,i) is poor. However, Ni©C can maintain 92% current density after the stability test for 120 h. Similarly, N─Ni©NC displays less loss of current density during the *I*‐*t* test, even at higher voltage (Figure S18, Supporting Information), which once again proves that the non‐fully encapsulated NC layer is vital for improving the stability of the N─Ni©NC.

In order to study the effect of different nitrogen sources on the electrocatalytic activity of N─Ni©NC, urea was replaced by dicyandiamide and melamine in the preparation process (Figure S19, Supporting Information). The results indicate that N doping from different nitrogen sources can enhance the HER and OER activity of the catalyst. The structural information of N─Ni©NC after HER and OER tests was investigated. After HER and OER testing, N─Ni nanosheets and non‐fully encapsulated NC layer structure are well preserved (Figures S20, S21, Supporting Information). The component of N─Ni©NC remains almost unchanged after HER test, but it undergoes surface reconstruction during OER, resulting in the formation of NiOOH species (Figures S21–S23, Supporting Information).^[^ ^45^ ^]^ Moreover, the concentration of residual Ni^2+^ in the alkaline electrolyte after the stability test is extremely low (Table S4, Supporting Information), attesting that N─Ni©NC does indeed have superb HER and OER stability.^[^ ^46^ ^]^

**Figure 5:**
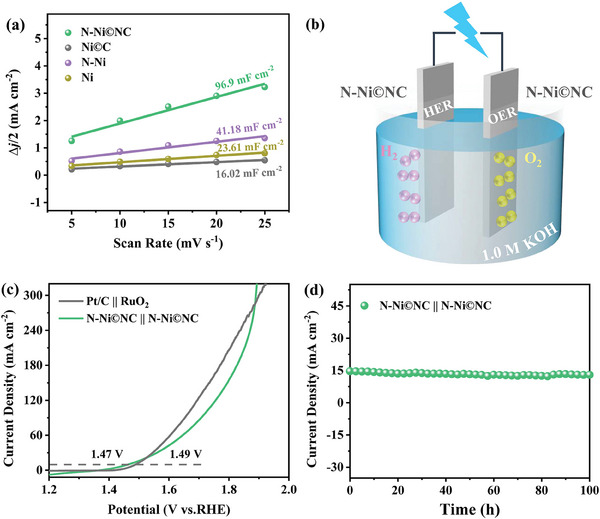
a) Electrochemical double layer capacitances of N─Ni©NC, Ni©C, N—Ni, and Ni. b) Schematic diagram of water electrolysis. c) LSV polarization curves of overall water splitting with N─Ni©NC || N─Ni©NC and Pt/C || RuO_2_. d) *I*‐*t* curve of N─Ni©NC || N─Ni©NC.

The electrochemical active surface area (ECSA) of the catalyst was determined by double‐layer capacitance (*C*_dl_,) which was calculated by testing the CV curve of non‐Faraday interval (Figure S24, Supporting Information). The *C*_dl_ value of N─Ni©NC is 96.9 mF cm^−2^ (**Figure** **5**a), which is higher than that of Ni©C, Ni, and N─Ni, verifying that N─Ni©NC possesses more accessible active sites. The ECSA‐normalized LSV curve (Figure S25, Supporting Information) was used to evaluate the specific catalytic activity of the catalyst. The results display that N─Ni©NC owns larger current density than the other samples, demonstrating its optimal catalytic activity. The turnover frequency (TOF) values were used to further estimate the intrinsic activity of catalysts (Figure S26, Supporting Information). N─Ni©NC has a higher TOF value than Ni©C at the overpotential of 100 (for HER) and 250 mV (for OER), indicating that the incorporation of N atom fundamentally alters the intrinsic activity of N─Ni©NC (Table S5, Supporting Information), which is conducive to efficient overall water splitting. N─Ni©NC free‐standing catalyst was employed directly as a cathode and anode to construct a water‐splitting electrolyzer (Figure 5b). As presented in Figure 5c, N─Ni©NC || N─Ni©NC delivers a current density of 10 mA cm^−2^ at a low cell voltage of 1.47 V, which is better than Pt/C || RuO_2_. In a 100 h *I*‐*t* testing, N─Ni©NC || N─Ni©NC exhibits excellent stability and great potential for practical water electrolysis (Figure 5d; Figure S27, Supporting Information).

### 2.3 Catalytic Mechanism

**Figure 6:**
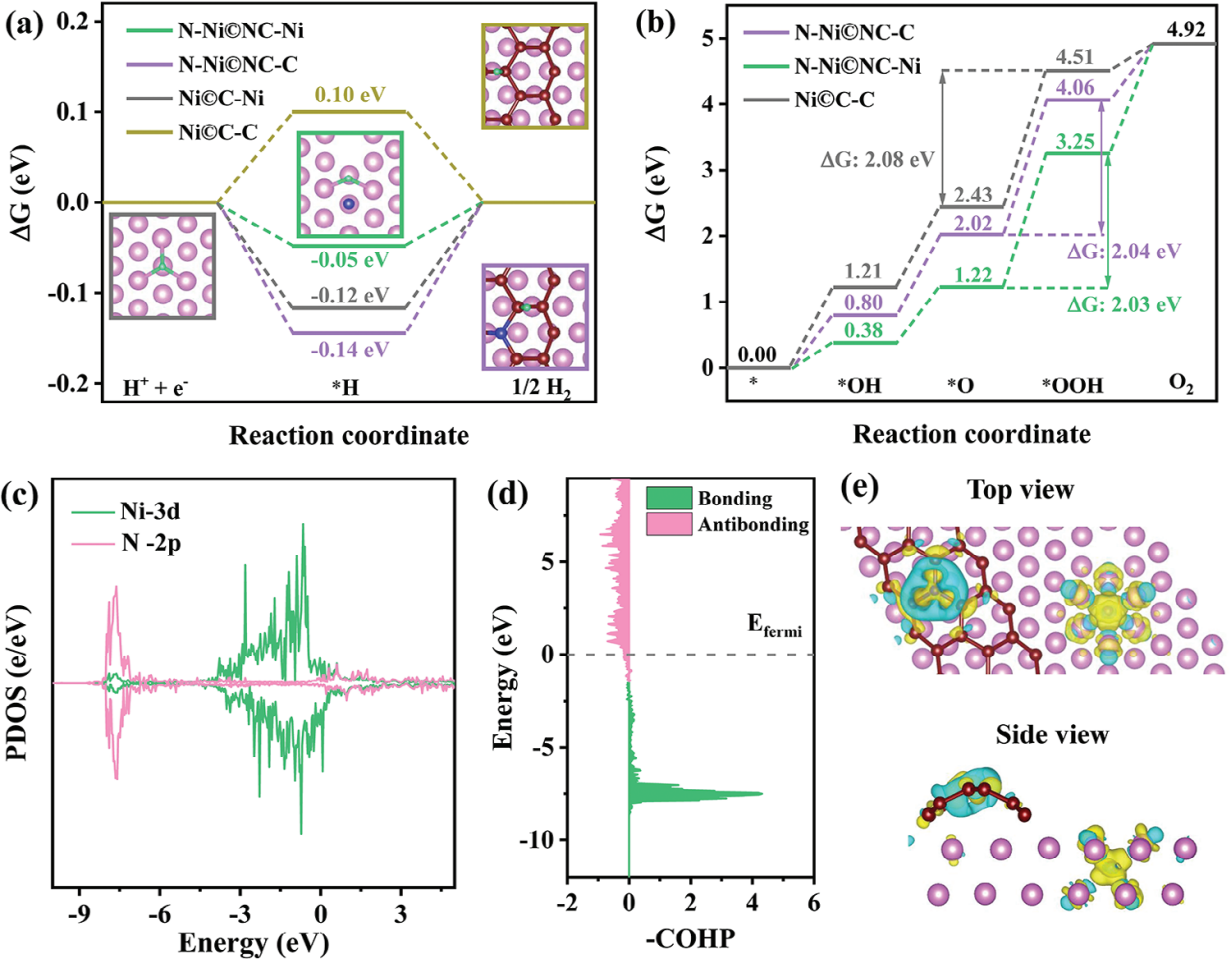
The Gibbs free energy diagrams of N─Ni©NC and Ni©C with different adsorption sites for a) HER and b) OER. c) PDOS plots of the N‐2p orbital and the Ni‐3d for N─Ni©NC. d) The COHP of Ni─N bond in N─Ni©NC. e) The charge density difference plots of N─Ni©NC. The blue and yellow isosurfaces stand for charge depletion and accumulation, respectively, in the space. The pink, dark red, blue, and green spheres correspond to Ni, C, N, and H atoms, respectively.

DFT calculations were conducted to uncover the role of incorporated N in enhancing HER and OER activity. Based on the TEM, XPS and XAFS results, the Ni crystal with surface non‐fully encapsulated by the C layer was used as the calculation model of Ni©C.^[^ ^47^ ^]^ Subsequently, one C atom is replaced by an N atom, while another N atom enters the body center of the Ni lattice accompanied by the generation of Ni vacancy, thus constructing the model for N─Ni©NC (Figure S28, Supporting Information). To determine the HER active sites of the catalysts, the Gibbs free energy change (ΔG_H*_) of hydrogen adsorption at different sites (Ni or C atoms) of N─Ni©NC and Ni©C were calculated. In **Figure** **6**a, the ΔG_H*_ value of Ni©C‐C (‐C refers to the adsorption site is C atom) is closer to 0 compared to that of Ni©C‐Ni (‐Ni stands for the adsorption site is Ni atom), manifesting that C in Ni©C is more inclined to be the active site of HER.^[^ ^48^ ^]^ When incorporated with N, the ΔG_H*_ values of N─Ni©NC‐Ni and N─Ni©NC‐C become ‐0.05 and ‐0.14 eV, respectively. The more suitable ΔG_H*_ value of N─Ni©NC‐Ni indicates that the incorporation of N makes Ni a highly efficient HER active site and greatly improves HER performance.^[^ ^43^ ^]^ ΔG_H*_ values of N─Ni©NC‐N and N─Ni©NC‐V_Ni_ (‐N and ‐V_Ni_ represent N and Ni vacancy as active sites) were also calculated (Figure S29a, Supporting Information). Obviously, ΔG_H*_ values of N─Ni©NC‐N and N─Ni©NC‐V_Ni_ are much larger than those of N─Ni©NC‐Ni, which further verifies that Ni as the active site in N─Ni©NC has the best hydrogen adsorption capacity.

To elucidate the association between the enhanced OER performance and the catalyst structure, the ΔG values of the four intermediates were investigated. The rate‐determining steps (RDS) for N─Ni©NC and Ni©C are both *O → *OOH due to their highest energy barriers (Figure 6b). The adsorption of *OOH at Ni for Ni©C is so weak that it cannot be used as the OER adsorption site, and only C can become the active site of OER with RDS energy barrier of 2.08 eV (Figure S30, Supporting Information).^[^ ^49^ ^]^ For N─Ni©NC, when Ni and C serve as adsorption sites respectively, their corresponding RDS energy barriers to form *OOH intermediates are 2.03 and 2.04 eV (Figures S31, S32, Supporting Information). This indicates that lattice N not only retains C as the active site but also activates the adsorption of OER intermediates by Ni. Consequently, the existence of dual active sites, both Ni and C, substantially boosts the catalytic efficiency of OER. The calculated partial density of states (PDOS) show a significant overlap between the N‐2p orbital and the Ni‐3d orbital, further indicating the bonding between Ni and N (Figure 6c). The crystal orbital Hamilton population (COHP) is used to evaluate the strength of Ni‐N bonds. Figure 6d displays that below the Fermi level, almost all states are bonded ones, which leads to more electrons occupying the bonded states, manifesting the strong bonding interaction between lattice N and Ni. The d‐band center position of Ni©C is −1.65 eV, while that of N─Ni©NC moves to −1.54 eV (Figure S34, Supporting Information), nearer the Fermi level. This proves that Ni transfers electrons to N through the strong Ni─N bonds, making it easier for oxygen‐containing intermediates to adsorb on partially positively charged Ni sites, further confirming that Ni sites are activated as OER active centers.^[^ ^50^ ^]^ Although the RDS energy barriers of N─Ni©NC‐Ni, N─Ni©NC‐C, and Ni©C‐C are close, there are two types of adsorption sites (Ni and C) in N─Ni©NC, but there is only one type of adsorption site (C) in Ni©C. Therefore, the OER performance of N─Ni©NC is preferable to that of Ni©C, which reasonably explains the experimental results. In addition, the ΔG values of N─Ni©NC‐N and N─Ni©NC‐V_Ni_ for OER intermediates were also calculated (Figures S29b, S33, Supporting Information). Among them, N hardly adsorbs *OH, and the RDS energy barrier of N─Ni©NC‐V_Ni_ is too large (2.89 eV), which is not beneficial to *OOH adsorption. In a word, the presence of Ni and C dual active sites induced by lattice N significantly promotes the electrocatalytic activity toward OER.

To understand the effect of introduced N atoms on the charge distribution, Bader charge analysis was performed. Due to the strong electronegativity of N, the charge numbers of C and Ni adjacent to N in N─Ni©NC are smaller than their counterparts in Ni©C (Figure S35, Supporting Information). This demonstrates that the introduction of N atom drives the charge redistribution of the surrounding atoms, thereby regulating the adsorption strength between the active sites and the reaction intermediates, and ultimately boosting the HER and OER activities.^[^ ^51^ ^]^ Figure 6e shows that in N─Ni©NC, electron transfers from Ni (and C) to N, which on the one hand makes high valence Ni the active site of OER, and on the other hand causes charge polarization of the atoms around N, thus enhancing the conductivity of the catalyst, which is consistent with the EIS results. The theoretical results suggest that lattice N activates Ni sites by regulating the electronic structure, thus enhancing the HER and OER intrinsic activities of the catalyst. This discovery provides important guidance for designing efficient electrocatalysts in the future.

## 3 Conclusion

In summary, a free‐standing electrode with N‐incorporated Ni nanosheets and non‐fully encapsulated by NC layer was constructed. The N─Ni©NC catalyst displays excellent HER and OER performance as well as ultra‐long stability. The bifunctional electrocatalyst only needs 1.47 V to achieve the current density of 10 mA cm^−2^ for overall water splitting. DFT calculations confirm that electrons transfer from Ni to N through the strong Ni─N bond, which makes the generated high valence Ni efficient active sites, thereby improving the OER activity of metallic Ni. It is essential to note that the incorporation of lattice N gives rise to the d‐band center closer to the Fermi level, which enhances the adsorption ability of Ni for *OOH and activates its OER activity. The presence of Ni and C dual active sites promotes the OER reaction kinetics and improves the catalytic efficiency. This work provides a feasible way for constructing efficient and robust free‐standing catalysts for water electrolysis by incorporating heteroatoms to activate the intrinsic activity of the host metal sites.

## Conflict of Interest

The authors declare no conflict of interest.

## Supporting information



Supporting Information

## Data Availability

The data that support the findings of this study are available from the corresponding author upon reasonable request.
