# Supplementary material for: Strong Bonding of Lattice N Activates Metal Ni to Achieve Efficient Water Splitting
Source: Adv Sci (Weinh). 2024 Nov 22;12(4):2411526. doi: 10.1002/advs.202411526 (PMC11789591; doi:10.1002/advs.202411526)
Supplement: Supplementary file 1 — Supporting Information [file ADVS-12-2411526-s001.docx]

**Supporting Information**

**Strong Bonding of Lattice N Activates** **Metal Ni to Achieve Efficient Water Splitting**

*Niandan Zhao, Wei Luo, Sijun Li, Hua Wang, Yini Mao, Yimin Jiang, Wenbin Wang*, Ming Li, Wei Su*, and Rongxing He**

Niandan Zhao, Wei Luo, Sijun Li, Hua Wang, Yini Mao, Yimin Jiang, Wenbin Wang, Ming Li, Rongxing He

Key Laboratory of Luminescence Analysis and Molecular Sensing (Southwest University), Ministry of Education, College of Chemistry and Chemical Engineering, Southwest University, Chongqing 400715, China

Email: [wwb20221720@swu.edu.cn](mailto:wwb20221720@swu.edu.cn) (W. Wang); [herx@swu.edu.cn](mailto:herx@swu.edu.cn) (R. He)

Wei Su

Key Laboratory of Beibu Gulf Environment Change and Resources Utilization (Nanning Normal University), Ministry of Education, College of Chemistry and Life Science, Nanning Normal University, 175 Mingxiu East Road, Nanning 530000, China

Email: [suw@nnnu.edu.cn](mailto:suw@nnnu.edu.cn) (W. Su)

**Experimental section**

**Materials**

There is no demand for additional purification because all of the reagents are analytical grade. Fuyang Bei Wood Pulp Paper Co., Ltd. in Hangzhou provided the cellulose. Nickel nitrate hexahydrate (Ni(NO_3_)_2_·6H_2_O) was supplied by Chengdu Kelong Chemical Co., Ltd. Potassium hydroxide (KOH), ethanol (C_2_H_5_OH) and sodium hydroxide (NaOH) were obtained from Chongqing Chuandong Chemical (Group) Co., Ltd. Nafion (5 wt%), urea (CH_4_N_2_O) and potassium thiocyanate (KSCN) were bought from Shanghai Aladdin Biochemical Technology Co., Ltd. Carbon paper (CP), Pt/C and RuO_2_ were ordered from Hesenbio Ltd. (China).

**Synthesis of** **cellulose/Ni(O****H)_2_ (Cel/Ni(OH)_2_), 0.5 cellulose/Ni(OH)_2_ and Ni(OH)_2_**

Cellulose (0.50 g) and Ni(NO_3_)_2_·6H_2_O (1.74 g) were added to deionized water (15 mL) and stirred to form slurry. The slurry was placed in the Teflon-lined autoclave for hydrothermal reaction (100 ℃ for 10 h) after its pH was raised to 10 by NaOH (1.5 mol L^-1^). To get Cel/Ni(OH)_2_, the sample was cooled to ambient temperature, then cleaned and dried in an oven at 60 °C. 0.5 cellulose/Ni(OH)_2_ was prepared by halving the amount of cellulose and keeping the other conditions unchanged. Ni(OH)_2_ was prepared by similar methods apart from not adding cellulose.

**Synthesis of N-Ni©NC, Ni©C, N-Ni©NC-0.5C, N-Ni@NC-3C and** **N-Ni@NC-5C**

N-Ni©NC was prepared by one step nitridation process. The porcelain boat was positioned in the upstream of the tube furnace and contained of urea (6.0 g), and Cel/Ni(OH)_2_ was positioned in the downstream. Reactants were heated to 500 ℃ for 2h in the Ar atmosphere (5 ℃ min^-1^), and N-Ni©NC-6 (abbreviated as N-Ni©NC) was obtained. Only the dosage of urea was changed to 0.0, 2.0, 4.0 and 8.0 g in the preparation process, and the final sample was labeled as N-Ni©NC-n (n = 0, 2, 4 and 8, where N-Ni©NC-0 is shortened to Ni©C). N-Ni©NC-0.5C was prepared by nitriding 0.5 cellulose/Ni(OH)_2_ under the same conditions. The synthesis of N-Ni@NC-3C and N-Ni@NC-5C was the same as N-Ni©NC, except that 0.25 g and 0.40 g cellulose were added in the calcination process, respectively.

**Synthesis of N-Ni and Ni**

The porcelain boat containing 6.0 g urea was placed in the upstream of the tube furnace, and Ni(OH)_2_ was placed in the downstream. In the Ar/H_2_ atmosphere, the furnace was heated to 500 ℃ (5 ℃ min^-1^) for 2 h, and N-Ni were synthesized. Only Ni(OH)_2_ was placed, calcined in the Ar/H_2_ atmosphere at 500 ℃ (5 ℃ min^-1^) for 2 h, and finally Ni was collected.

**Poisoning experiments**

The N-Ni©NC (or Ni©C) catalyst was immersed in KSCN solution (50 mM) at room temperature for 24 h. Then the catalyst was washed and dried, and the SCN^-^ poisoned N-Ni©NC (or Ni©C) catalyst was obtained, labeled as N-Ni©NC+SCN^-^ (or Ni©C+SCN^-^).

**Characterizations**

The morphology and microstructure were analyzed by scanning electron microscope (SEM, FlexSEM 1000II, Japan) and high-resolution transmission electron microscopy (HRTEM, FEI Talos F200x, USA). The crystal phases of catalysts were investigated using X-ray diffraction (XRD, Bruker D2 PHASER, Germany) with Cu *Kα* radiation. The chemical compositions were collected by X-ray photoelectron spectroscopy (XPS, Thermo Scientific K-Alpha, USA). Raman spectra were measured by Raman spectrometer (LabRAM HR Evolution, Japan). The EPR spectroscopy was identified via Electron Paramagnetic Resonance Spectrometer (Bruker EMXplus-6/1, Germany). The surface area was recorded by nitrogen adsorption/desorption analysis (BET, Micromeritics ASAP 2460, USA). Quantitative analysis of ion was achieved by the ICP-OES (Agilent 5110(OES), USA). The contact angle (CA) measurement was carried out in JC2000D (Powereach Co., Shanghai, China). The X-ray absorption fine structure spectra (XAFS, Fe K-edge) were measured at 1W1B station in Beijing Synchrotron Radiation Facility (BSRF) in ambient conditions.

**Electrochemical tests**

Electrochemical tests were carried out on CHI 760 electrochemical workstation and the Autolab PGSTAT-302N workstation. In the three-electrode system, N-Ni©NC was used as the working electrode, Hg/HgO electrode was used as the reference electrodes in 1.0 M KOH electrolytes, graphite rod was served as the counter electrode. For the preparation of the comparison electrode, a mixture of 580 μL ethanol, 400 μL deionized water and 20 μL Nafion was employed to disperse 10 mg sample. Sonicated until a uniform ink was formed, 0.5 mL catalyst ink was distributed evenly onto 1 × 1 cm^2^ CP, and dried for use. The linear sweep voltammetry (LSV) curve was recorded at a scanning rate of 5 mV s^-1^ (90% IR compensation). The electrochemical double-layer capacitance (*C*_dl_) was measured by testing CV curves at different scan rates (5, 10, 15, 20 and 25 mV s^-1^). The electrochemical impedance spectroscopy (EIS) was conducted in the frequency range of 10^5^ to 0.1 Hz. The durability of catalyst was evaluated by chronoamperometry method. The value of turnover frequency (TOF) was determined based on the following formula: $TOF=\frac{I}{mFn}$, where $I$, $m$, and $F$ are the current, the number of electrons required for the water to produce one H_2_ (m = 2) or O_2_ (m = 4), Faraday’s constant, respectively. $n$ is the number of active sites that can be obtained by $n=\frac{Q}{2F}$. Assuming it is one electron redox process, $Q$ represents the total integrated charge in the potential range of CV curve performed in PBS solution. All potentials were converted to reversible hydrogen electrode (RHE) by the following formula:

$$E_{RHE}=E_{Hg/HgO}+0.0591\times pH+0.098$$

$$E_{RHE}=E_{Ag/AgCl}+0.0591\times pH+0.197$$

**Computational details**

Density functional theory (DFT) was used to perform relevant calculations on catalysts and their reaction intermediates on Vienna *ab initio* simulation package (VASP). The electron-ion interaction was depicted using projector-augmented wave (PAW) pseudopotential. The generalized gradient approximation (GGA) and the Perdew-Burke-Ernzerhof (PBE) functional were applied.^[1-5]^ The cut-off energy was 400 eV. 2 × 2 × 1 was chosen as the Monkhorst-Pack grid k-points in the Brillouin zone during the structural optimization. The total energy was converged to 1 × 10^-5^ eV and the residual force on each relaxed atom was less than 0.02 eV Å^−1^. The vacuum layer was selected to 15 Å along the c-axis to prevent periodic interactions. The composite was constructed using 3×3 Ni (111) and 3×6 graphene (001). The thickness of the Ni (111) slab was determined to be three layers, and the lowest layer of Ni (111) was fixed to simulate the bulk atoms during the calculation.


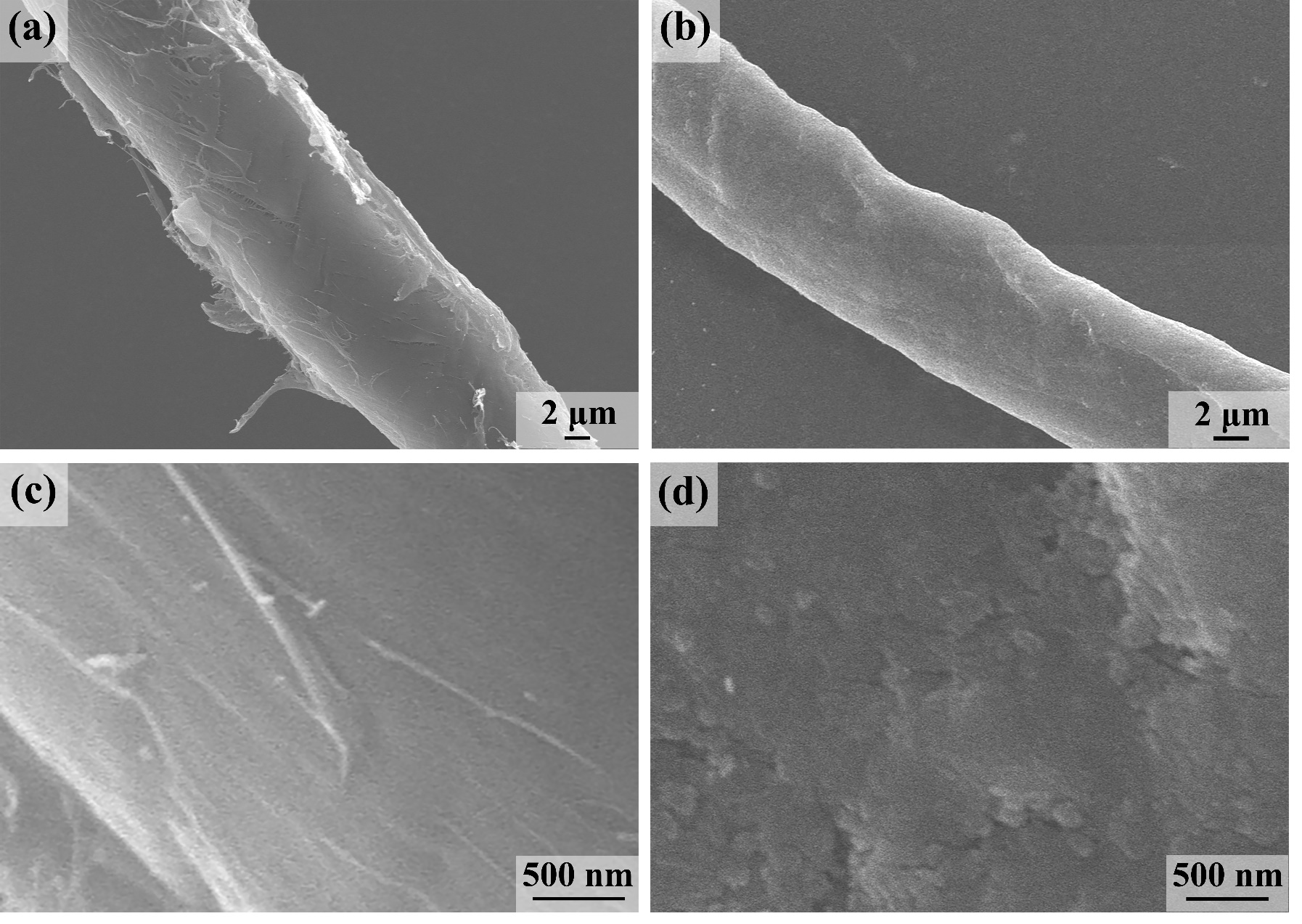


**Figure S1.** SEM images of (a, c) Cellulose and (b, d) Cel/Ni(OH)_2_.


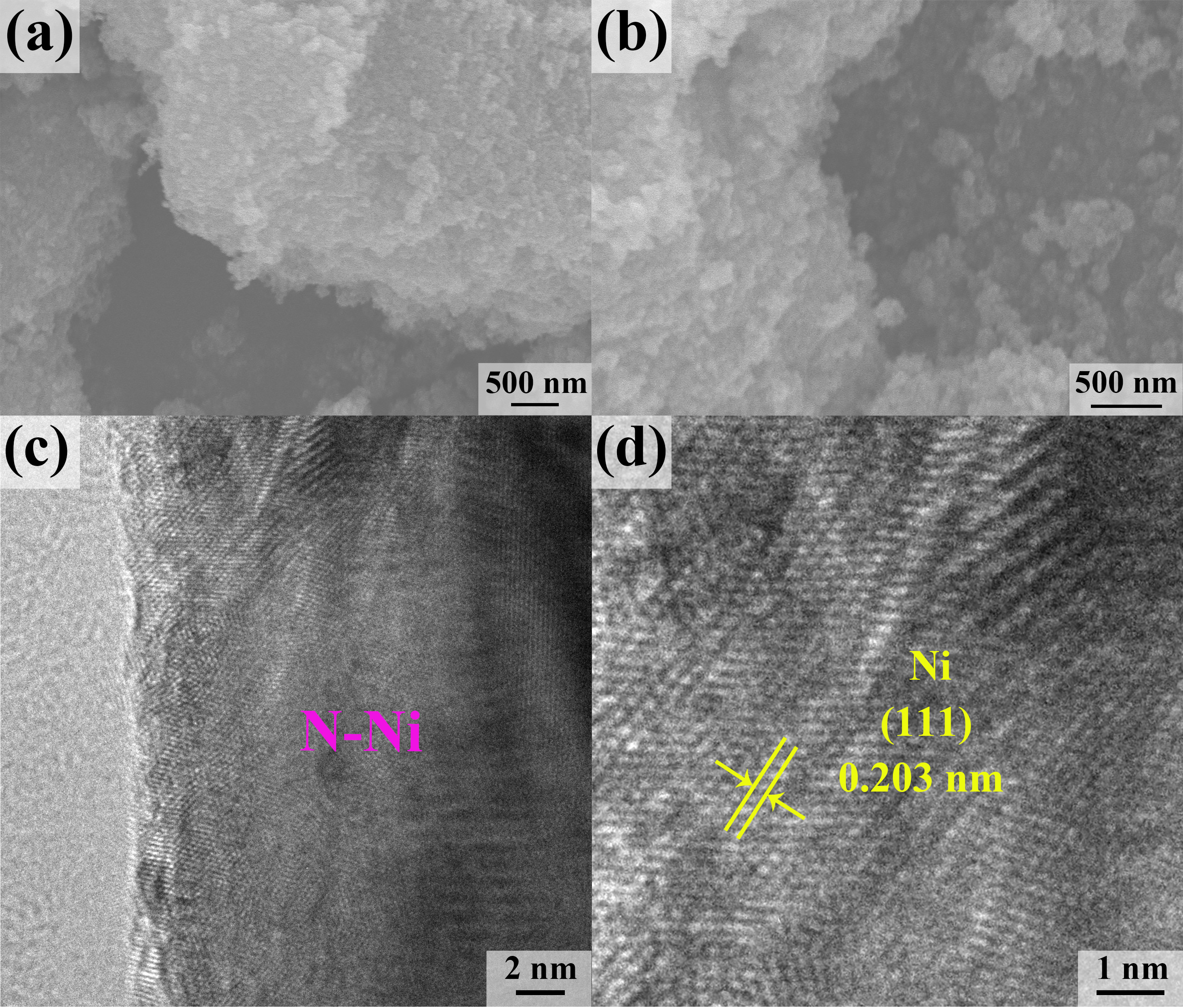


**Figure S2.** (a, b) SEM and (c, d) TEM images of N-Ni.


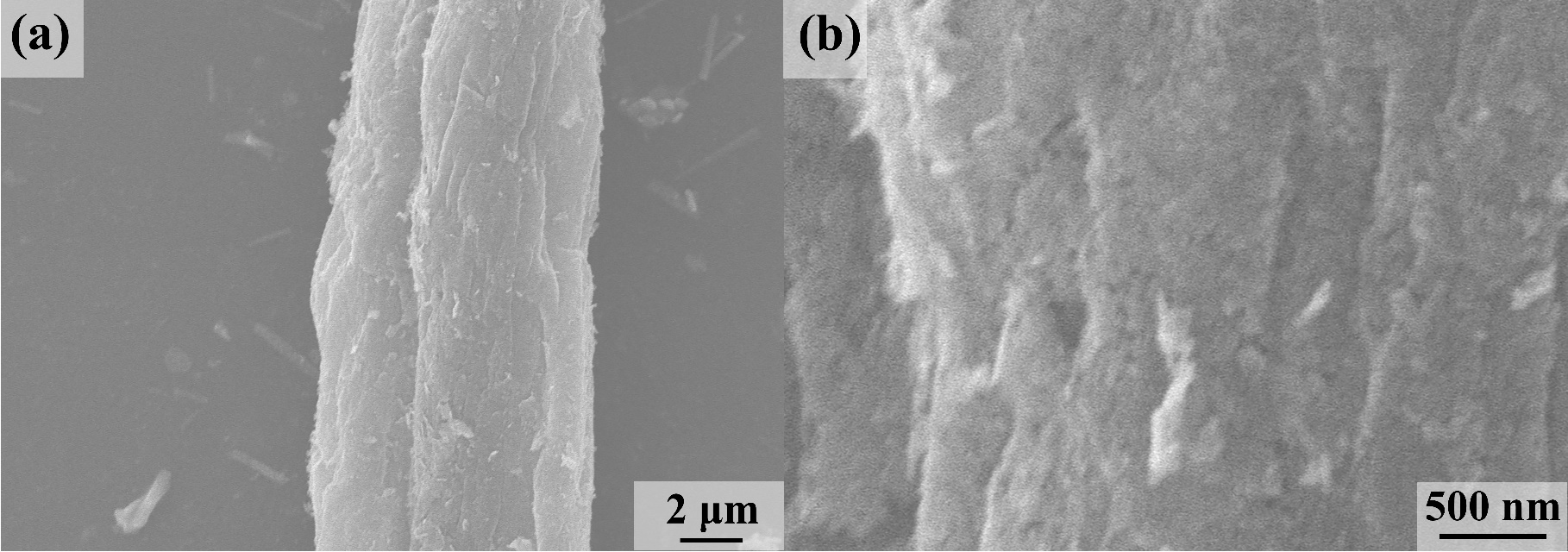


**Figure S3.** SEM images of N-Ni©NC.


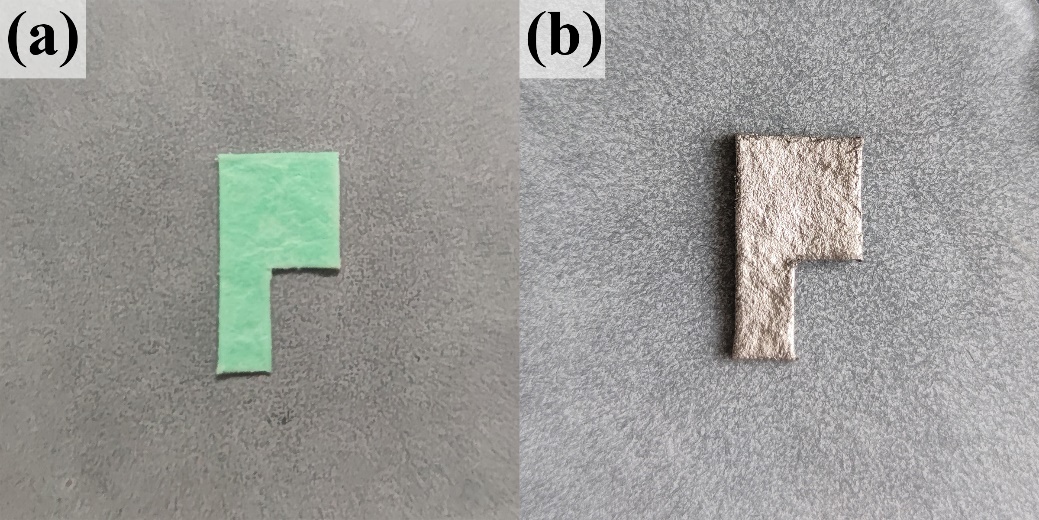


**Figure S4.** Photos of (a) Cel/Ni(OH)_2_ and (b) N-Ni©NC electrode.

**Figure S5.** XRD patterns of the products obtained by calcining Cel/Ni(OH)_2_, Ni(OH)_2_ and Cel + Ni(OH)_2_ at 500 ℃ for 2 h under Ar atmosphere.

Cellulose plays a vital role in the construction of N-Ni©NC free-standing electrocatalysts. First of all, using cellulose or its derivatives (carbon fiber) as the substrate can directly constitute the free-standing electrode, and the photos of Cel/Ni(OH)_2_ precursor and N-Ni©NC electrode are shown in Figure S4. Secondly, during the pyrolysis process of cellulose, Ni(OH)_2_ is reduced to Ni by the C-containing gas, which also acts as seeds to catalyze the growth of the carbon layer. Thirdly, if cellulose and Ni(OH)_2_ are physically mixed (marked as Cel + Ni(OH)_2_) and calcined under the same procedure, Ni/NiO/C is obtained (Figure S5), manifesting that Ni(OH)_2_ grown in situ on cellulose is more conducive to complete reduction.


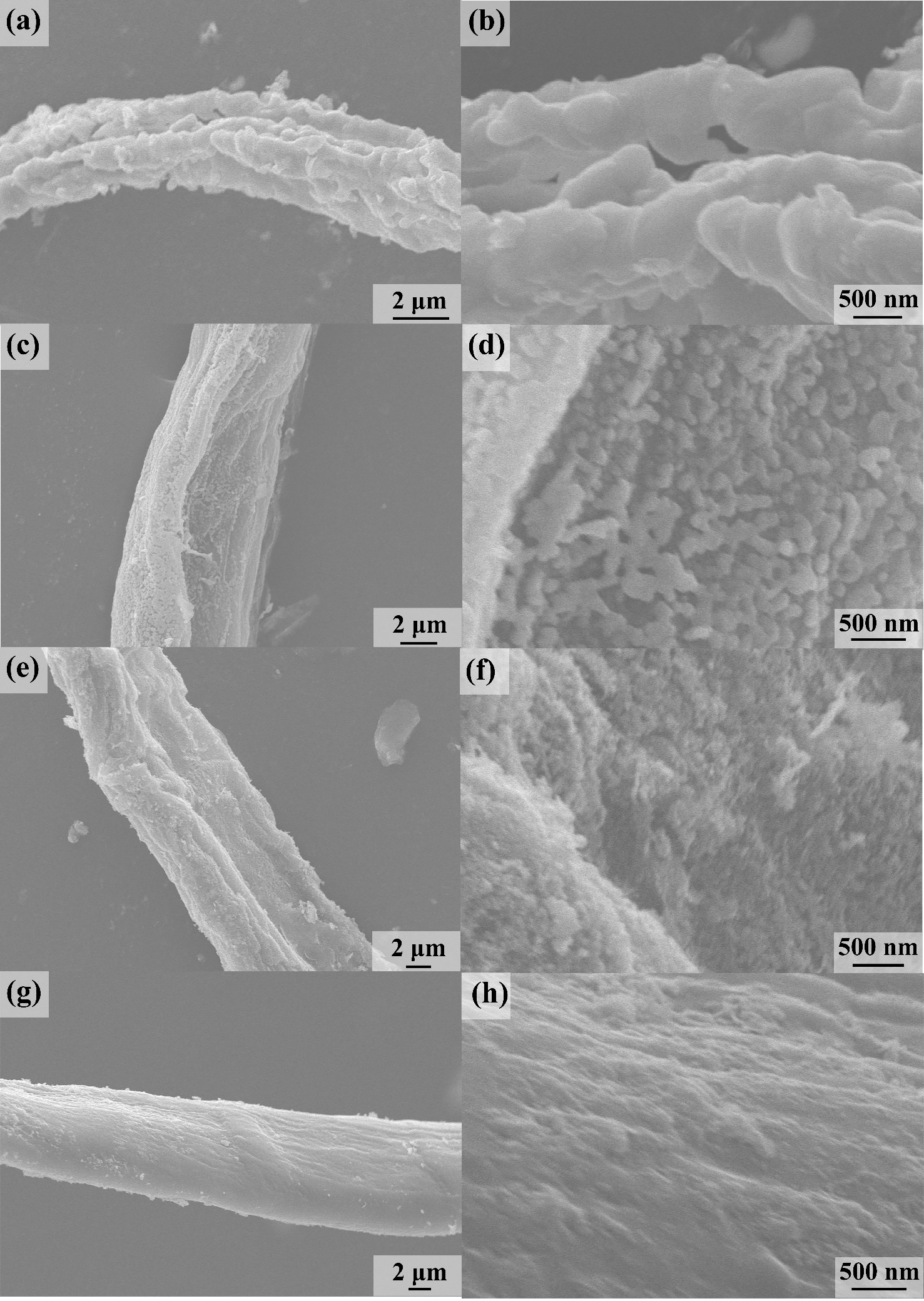


**Figure S6.** SEM images of (a, b) Ni©C, (c, d) N-Ni©NC-2, (e, f) N-Ni©NC-4 and (g, h) N-Ni©NC-8.

**Figure S7.** The nitrogen adsorption/desorption isotherms of Ni©C and N-Ni©NC.


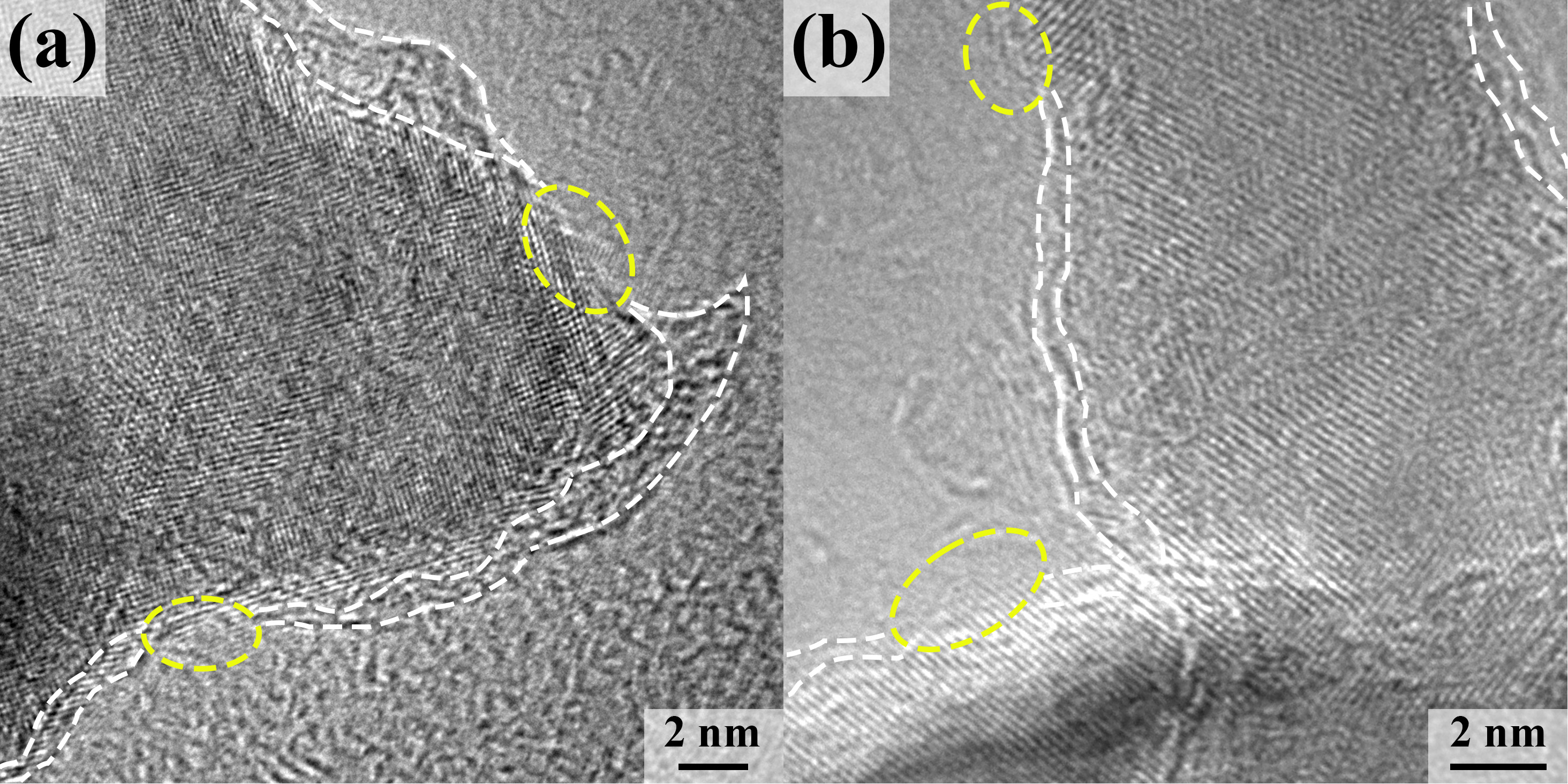


**Figure S8.** TEM images of N-Ni©NC.

**
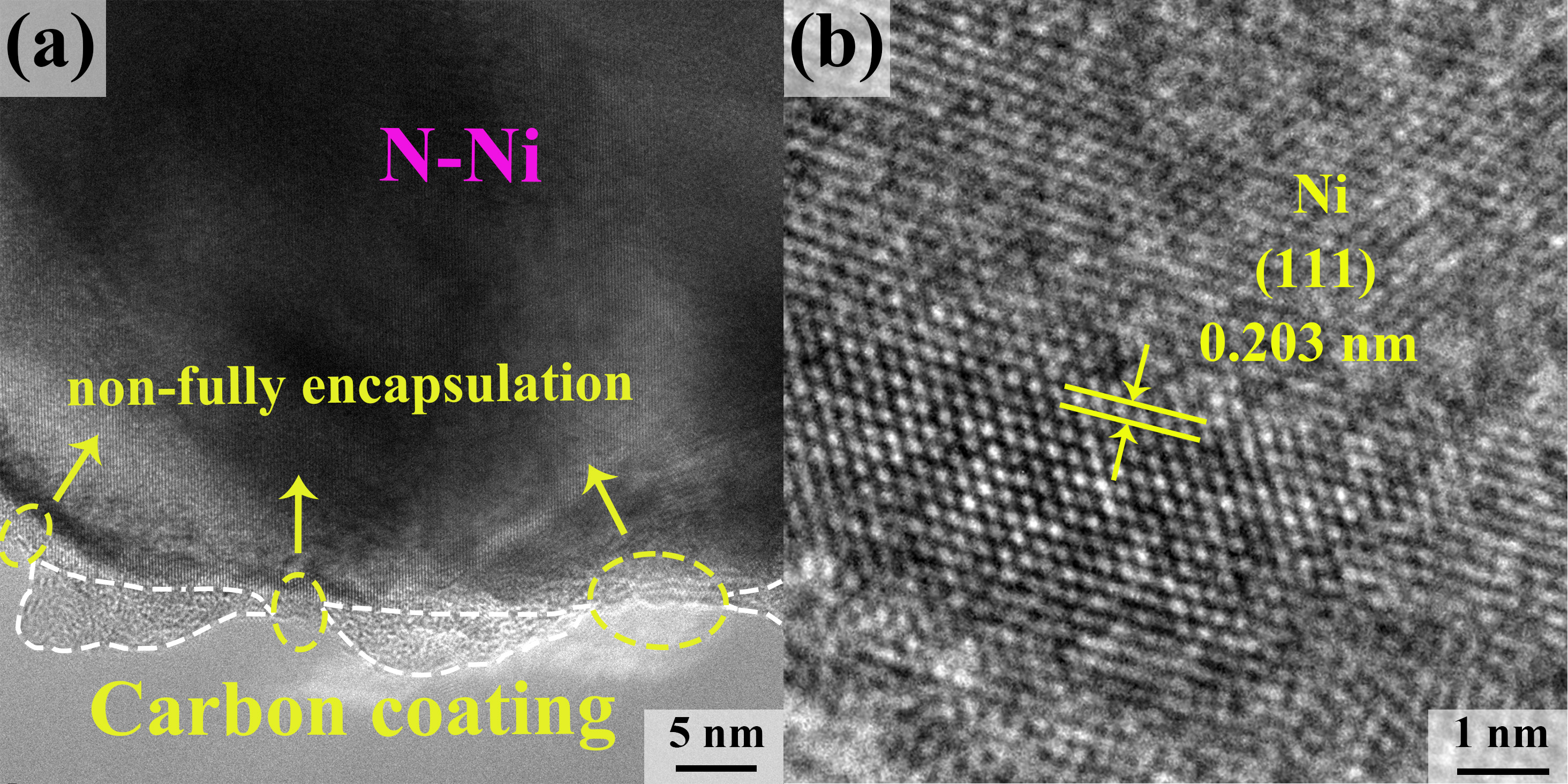
**

**Figure S9.** TEM images of N-Ni©NC-0.5C.

**Figure S10.** XRD patterns of the (a) Cellulose and Cel/Ni(OH)_2_, along with (b) N-Ni©NC-n (n = 2, 4, 6 and 8).

Figure S10a shows the diffraction peak at 2θ of 22.4^o^ in the Cel/Ni(OH)_2_ precursor is assigned to the (002) plane of cellulose, and the remaining peaks are attributed to the characteristic diffraction peaks of Ni(OH)_2_ (PDF#14-0117).

In Figure S10b, as the concentration of lattice N increases, the diffraction peaks of Ni gradually widen, indicating that the N atom affects the crystallinity of the material.

**Figure S11.** XPS spectra of N-Ni©NC and Ni©C, (a) survey, (b) C1s and (c) N 1s.

**Figure S12.** EPR spectra of N-Ni©NC and Ni©C.

**Figure S13**. (a) HER polarization curves and (b) Tafel plots of N-Ni©NC-n (n = 2, 4, 6 and 8) in 1.0 M KOH, (c) Overpotential histogram at current density of 10, 50 and 100 mA cm^−2^.


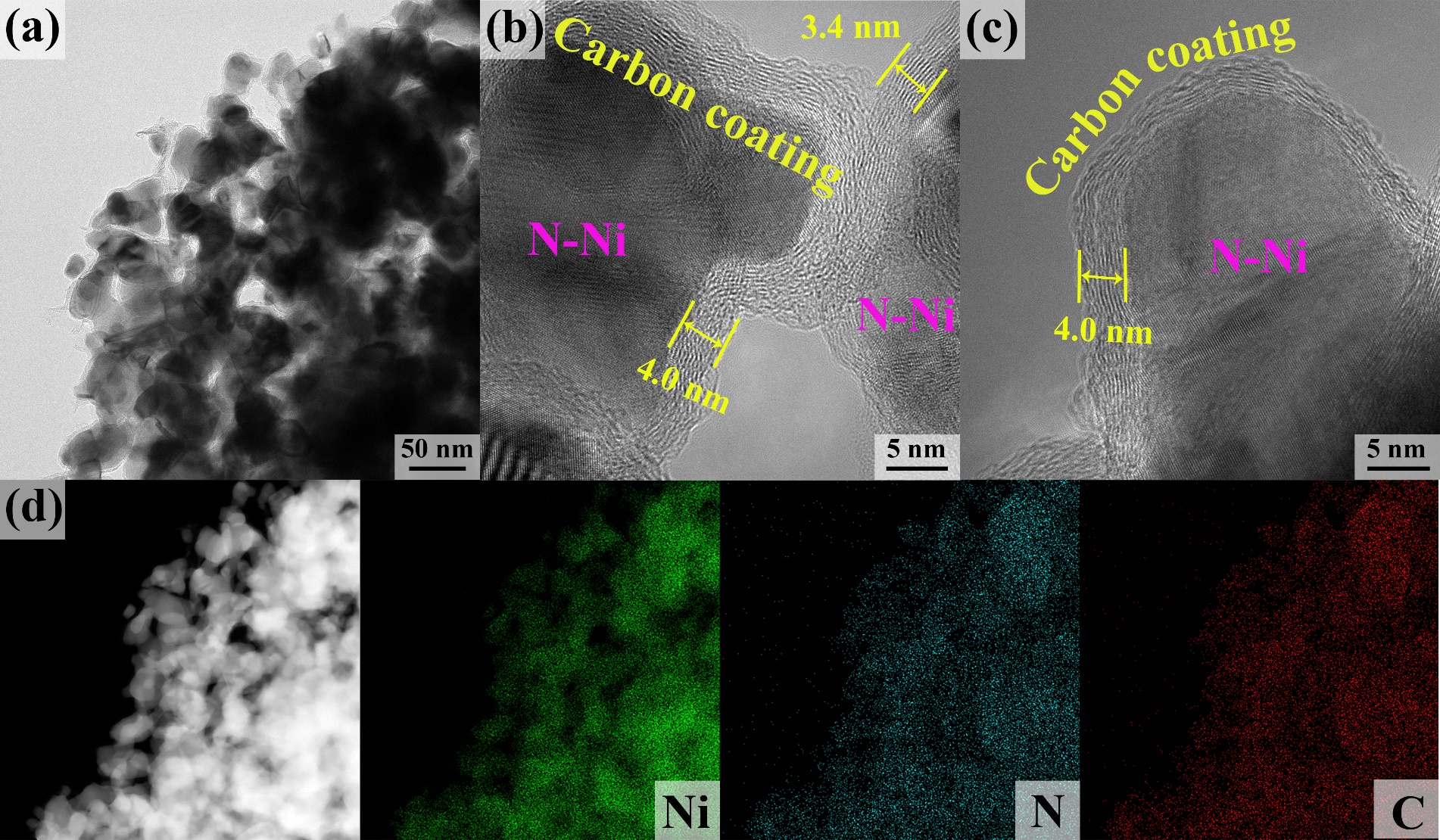


**Figure S14**. (a-c) TEM and HRTEM images, (d) HAADF-STEM image and EDS-mapping pictures of N-Ni@NC-3C.


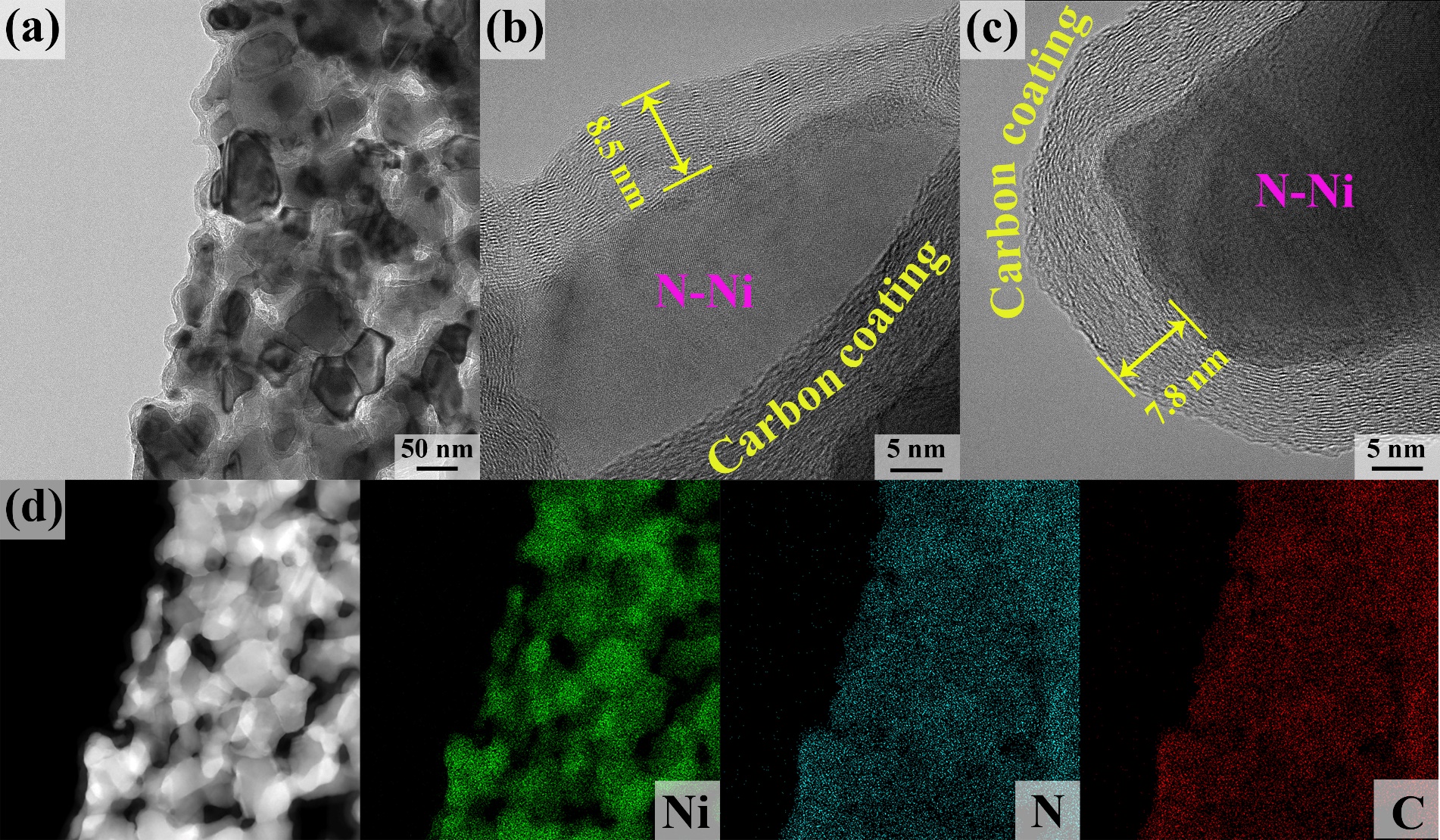


**Figure S15**. (a-c) TEM and HRTEM images, (d) HAADF-STEM image and EDS-mapping pictures of N-Ni@NC-5C.

**Figure S16**. *I*-*t* curve of N-Ni©NC for HER.

**Figure S17**. (a) OER polarization curves and (b) Tafel plots of N-Ni©NC-n (n = 2, 4, 6 and 8) in 1.0 M KOH, (c) Overpotential histogram at current density of 10, 20 and 50 mA cm^−2^.

**Figure S18**. *I*-*t* curve of N-Ni©NC for OER.

.

**Figure S19**. XRD patterns of N-Ni©NC, N-Ni©NC-Dicyandiamide and N-Ni©NC-Melamine. (b) HER and (c) OER polarization curves of N-Ni©NC, N-Ni©NC-Dicyandiamide and N-Ni©NC-Melamine.

N-Ni©NC-Dicyandiamide and N-Ni©NC-Melamine were prepared by replacing the nitrogen source with dicyandiamide and melamine, respectively. As can be seen from Figure S19a, changing different nitrogen sources does not affect the composition of the catalyst. In addition, all three catalysts exhibit excellent HER and OER properties regardless of the nitrogen source, indicating that the catalytic performance was less affected by the type of nitrogen source (Figures S19b and 19c).


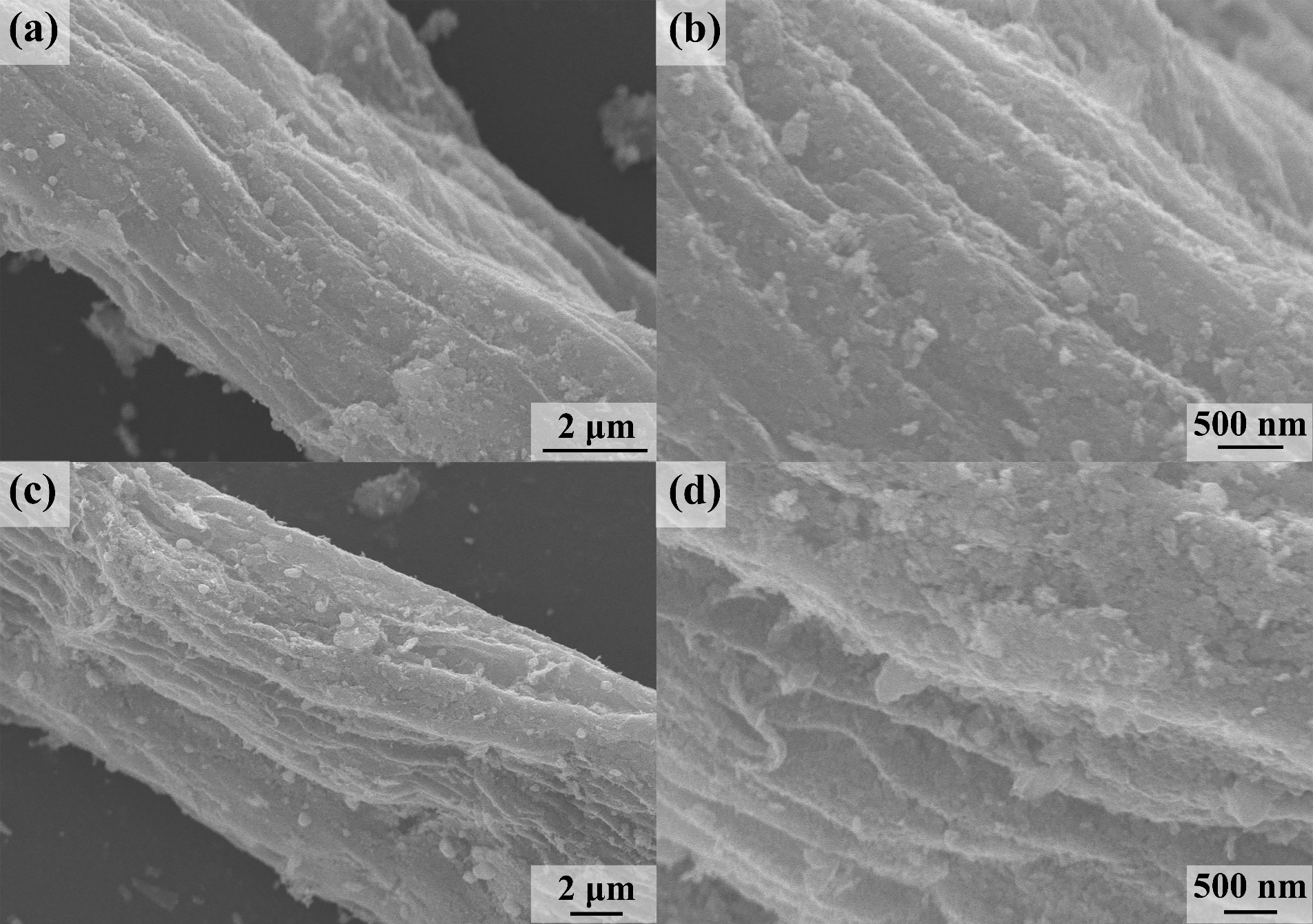


**Figure S20.** SEM images of N-Ni©NC after (a, b) HER and (c, d) OER.


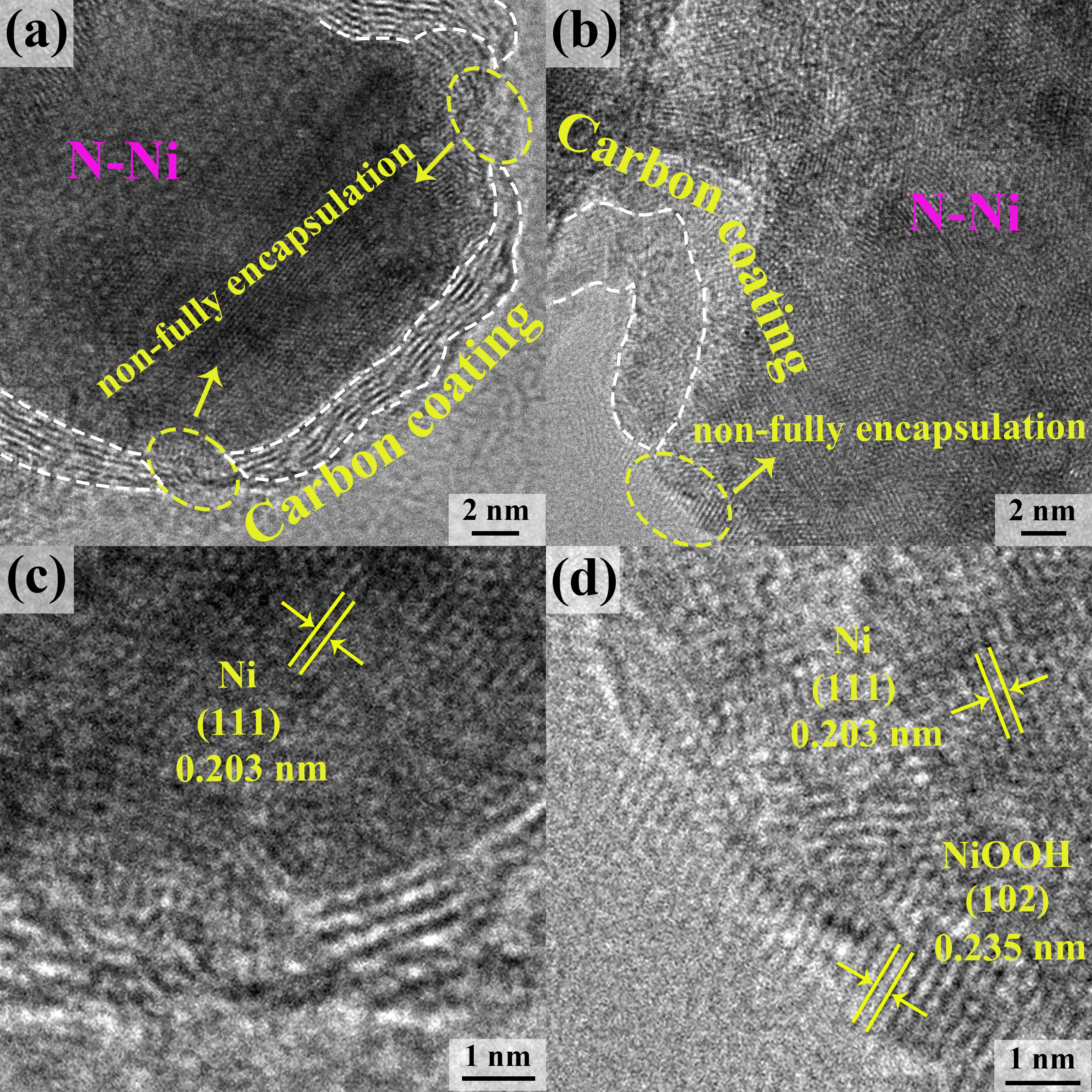


**Figure S21**. TEM images of N-Ni©NC after (a, c) HER and (b, d) OER.

**

**

**Figure S22.** (a) C 1s, (b) Ni 2p and (c) N 1s XPS spectra of N-Ni©NC before and after HER stability tests.





**Figure S23.** (a) C 1s, (b) Ni 2p and (c) N 1s XPS spectra of N-Ni©NC before and after OER stability tests.

**Figure S24.** Cyclic voltammogram curves of (a) N-Ni©NC, (b) Ni©C, (c) N-Ni and (d) Ni at different scan rates (5, 10, 15, 20 and 25 mV s^-1^).

**Figure S25.** Polarization curves normalized by ECSA of N-Ni©NC, Ni©C, N-Ni and Ni for (a) HER and (b) OER.

**Figure S26.** Cyclic voltammetry curves of (a) N-Ni©NC and (b) Ni©C were measured at 0.05 V s^-1^ in phosphate buffered saline solution (PBS, pH = 7.0).

**Figure S27.** *I*-*t* curve of N-Ni©NC || N-Ni©NC for overall water splitting.


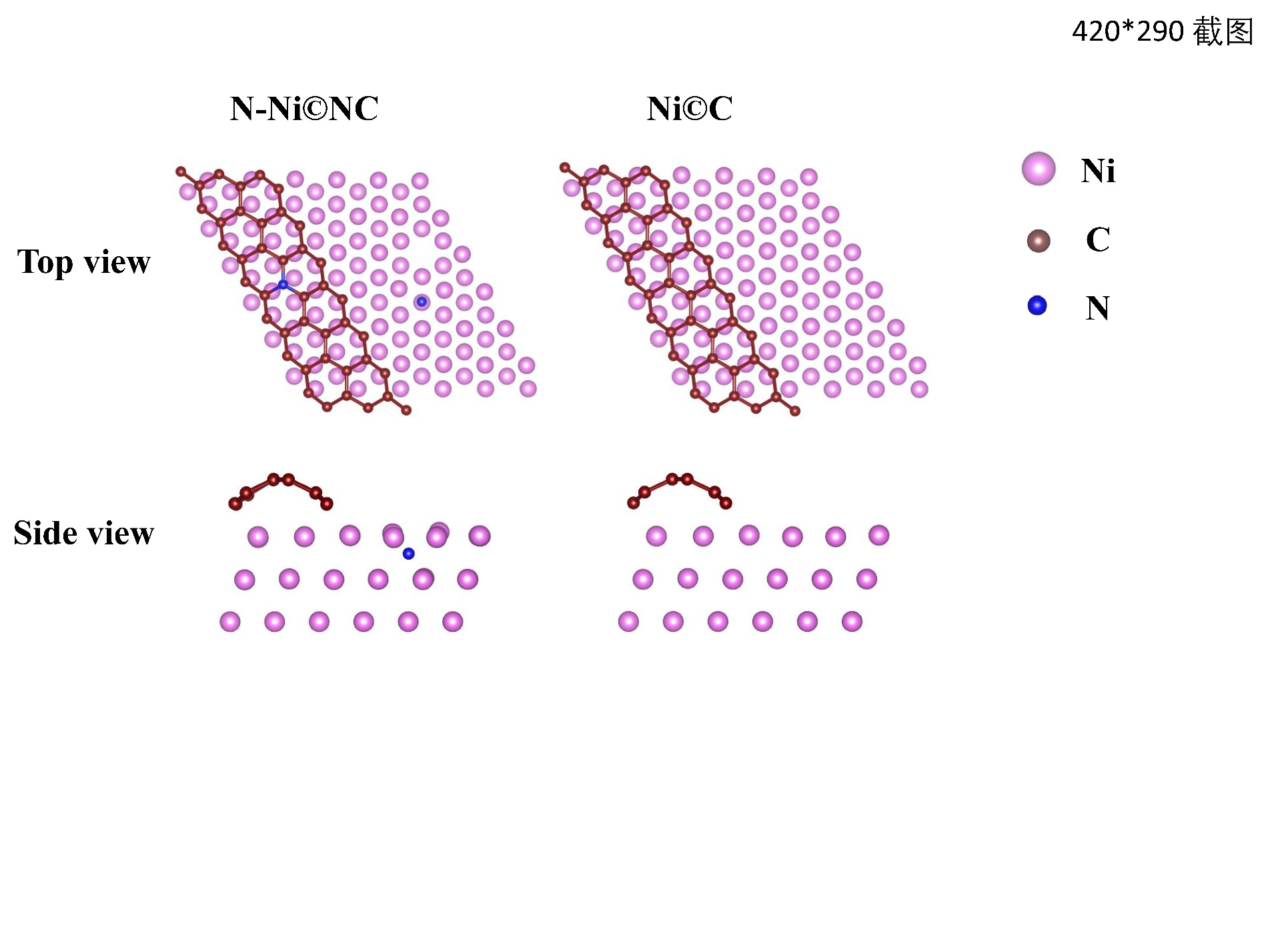


**Figure S28.** Optimized models of N-Ni©NC and Ni©C.

**Figure S29.** The Gibbs free energy diagrams of N-Ni©NC and Ni©C with different adsorption sites for (a) HER and (b) OER.


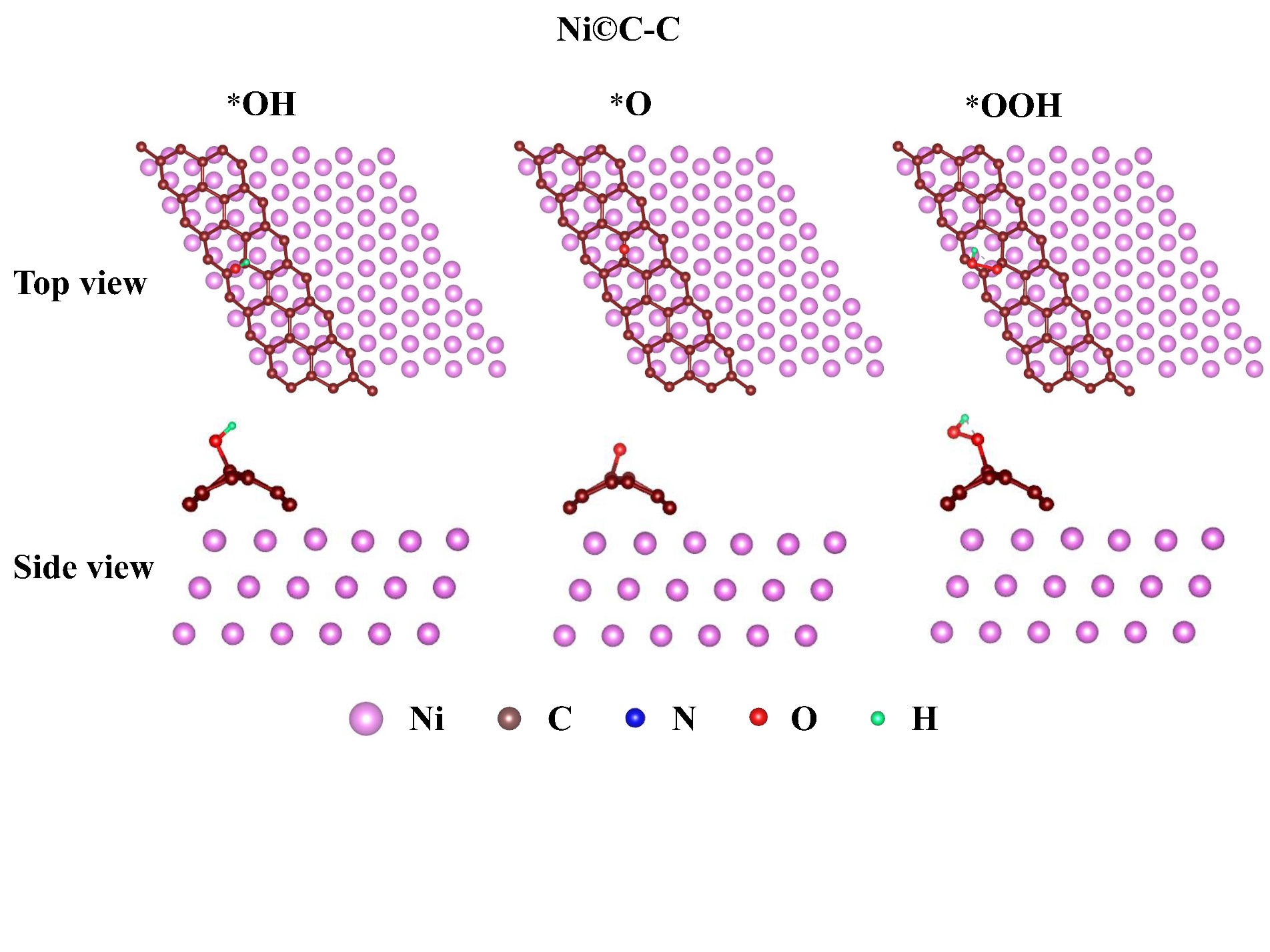


**Figure S30.** Optimized configurations of OER intermediates on Ni©C with C site.


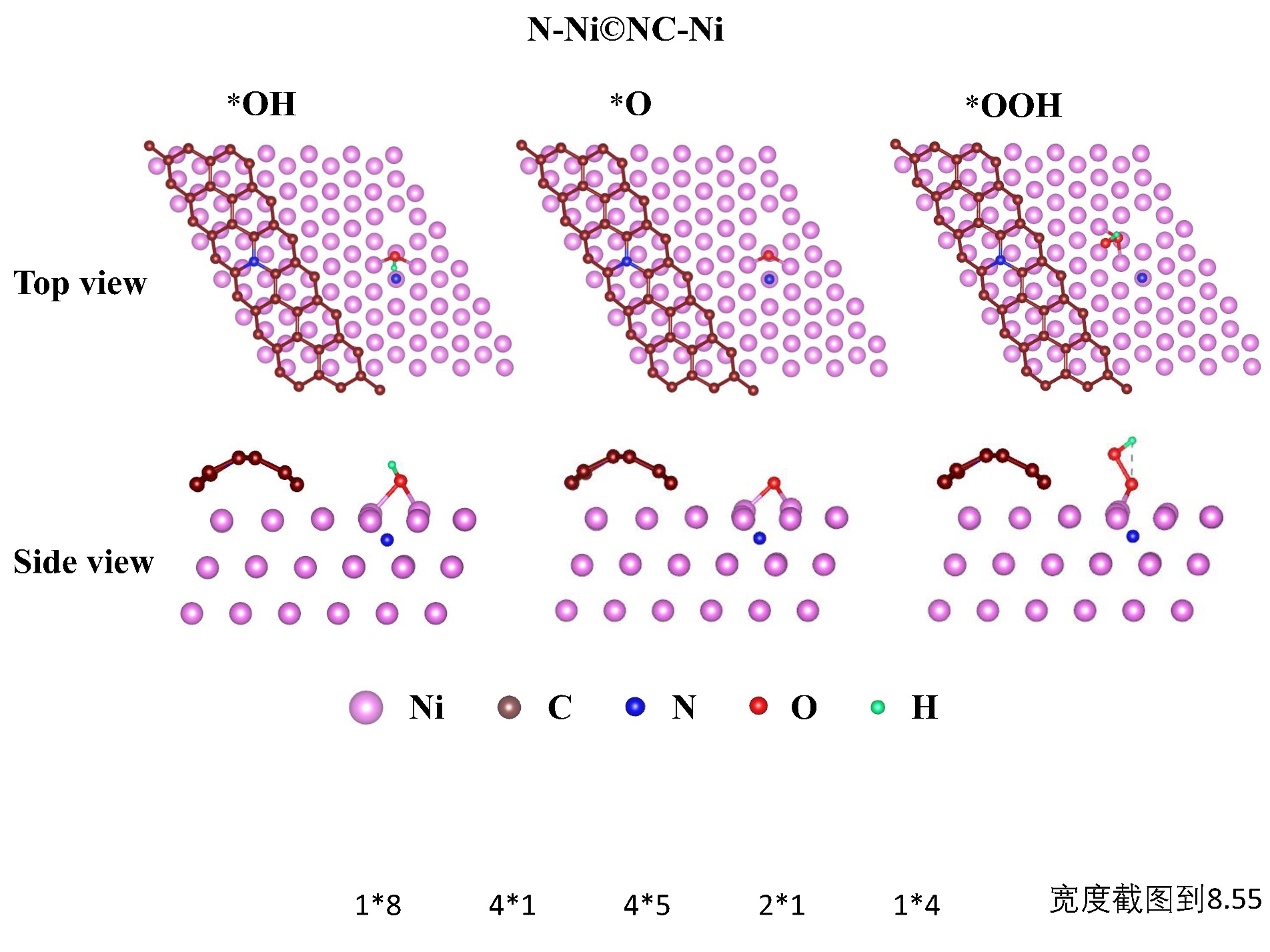


**Figure S31.** Optimized configurations of OER intermediates on N-Ni©NC with Ni sites.


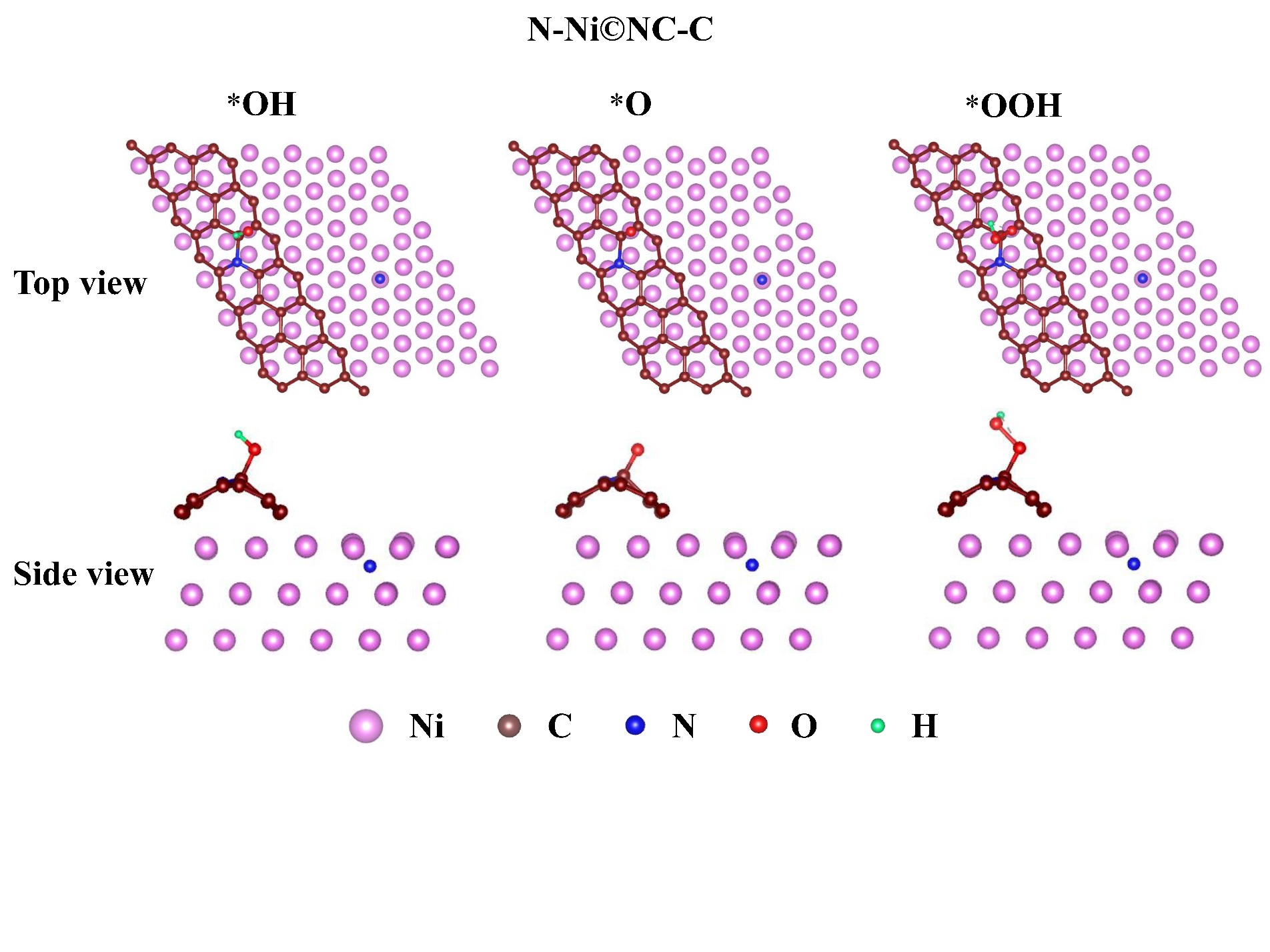


**Figure S32.** Optimized configurations of OER intermediates on N-Ni©NC with C sites.


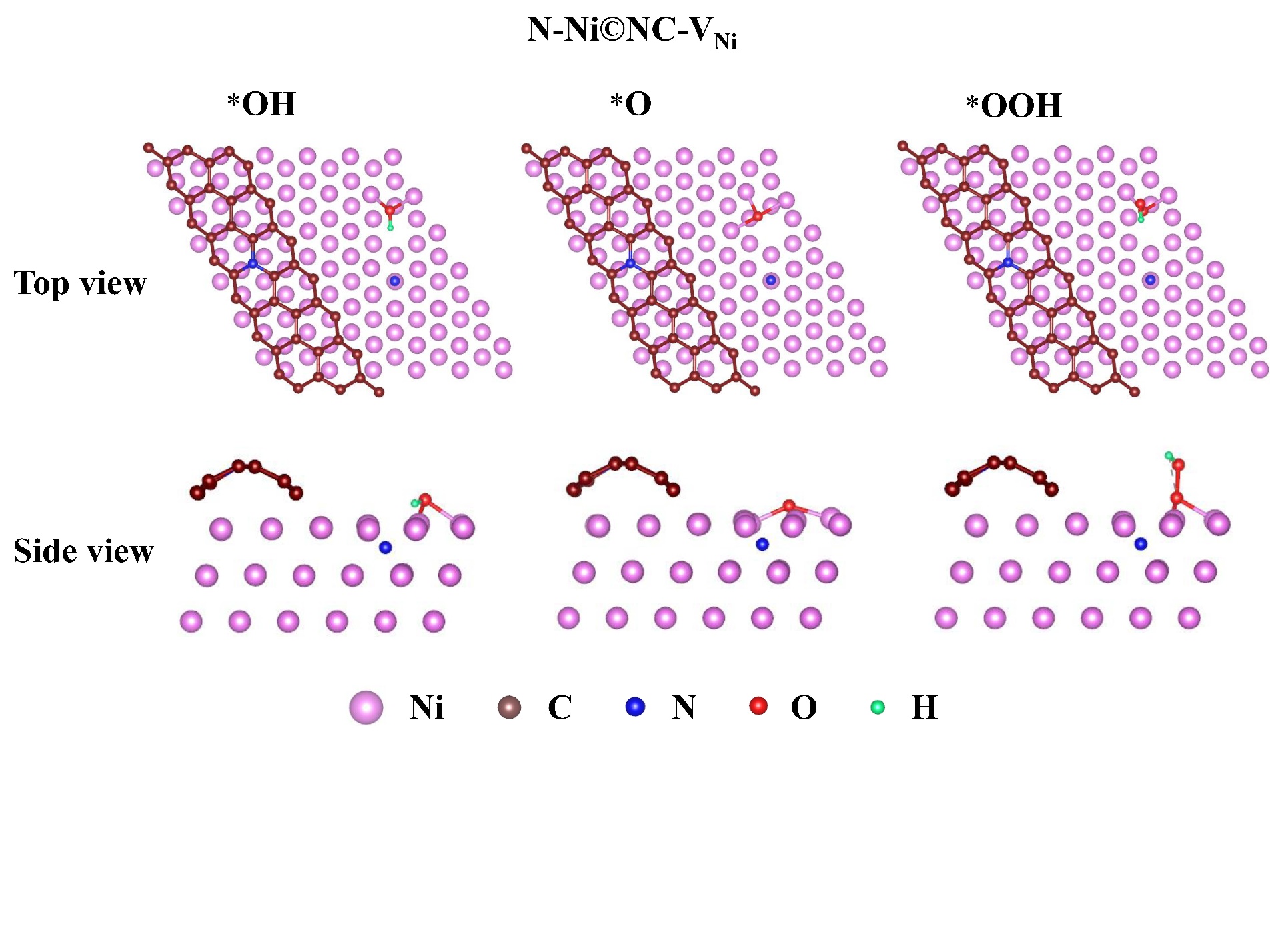


**Figure S33.** Optimized configurations of OER intermediates on N-Ni©NC with V_Ni_ sites.

**Figure S34.** The d-band center position of N-Ni©NC and Ni©C.


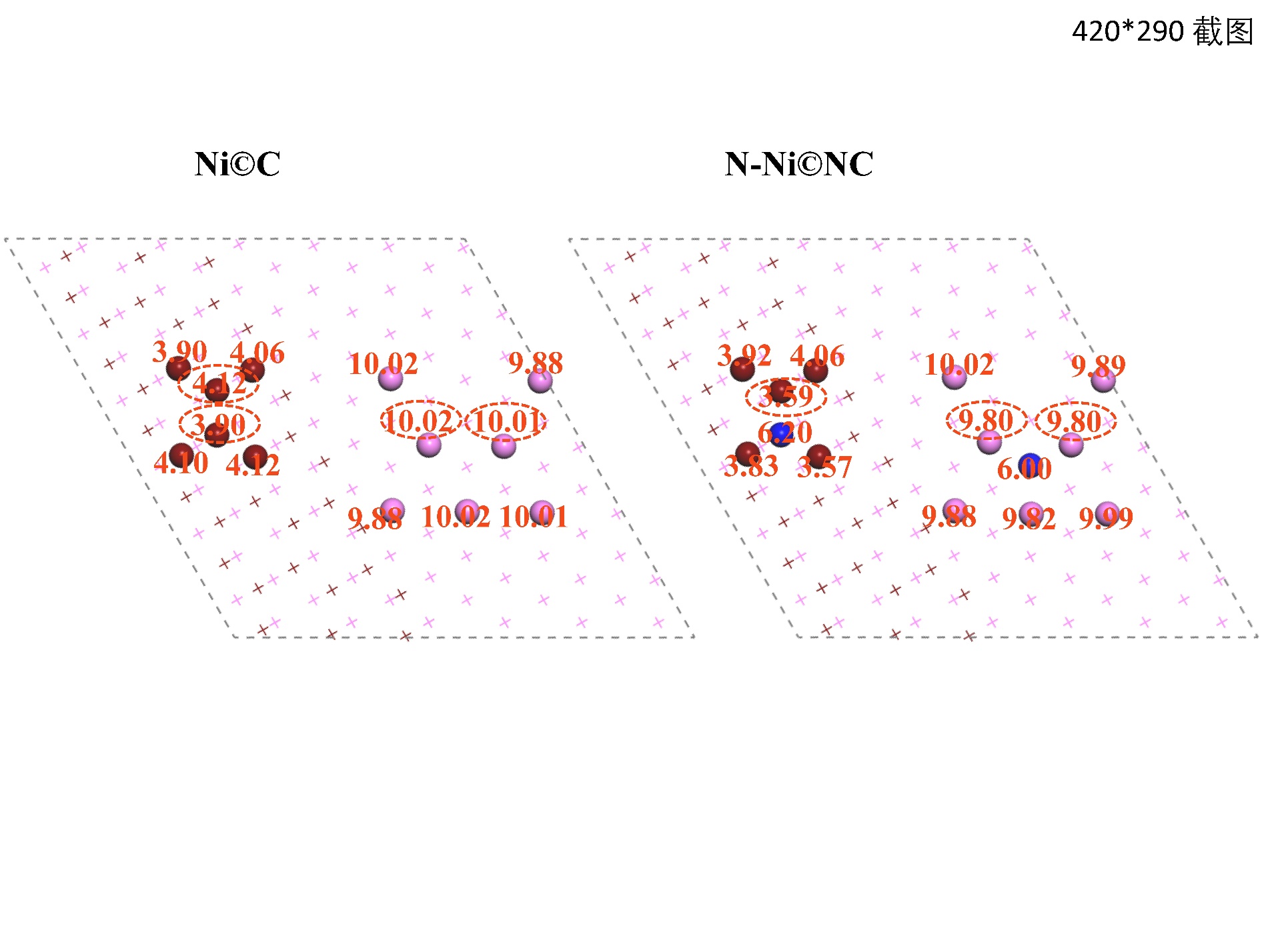


**Figure S35.** The Bader charge numbers of atoms in Ni©C and N-Ni©NC (Orange dotted circles represent adsorption sites).

**Table S1.** EXAFS fitting parameters at the Ni *K*-edge for N-Ni©NC, Ni©C, Ni foil and NiO.

| Sample | Shell | N ^a^ | R (Å) ^b^ | σ^2^ (Å^2^·10^-3^) ^c^ | ΔE_0_ (eV) ^d^ | *R* factor (%) |
| --- | --- | --- | --- | --- | --- | --- |
| N-Ni©NC | Ni-N | 0.4 | 1.81 | 6.7 | -5.1 | 0.8 |
|  | Ni-Ni | **8.2** | 2.48 | 5.4 | 5.1 |  |
| Ni©C | Ni-O | 0.3 | 1.95 | 4.3 | 3.1 | 0.3 |
|  | Ni-Ni | **9.1** | 2.48 | 5.6 | 6.6 |  |
| NiO | Ni-O | 6 | 2.10 | 6.4 | 0.8 | 0.8 |
|  | Ni-O-Ni | 12 | 2.95 | 6.2 | -4.2 |  |
| Ni foil | Ni-Ni | 12 | 2.48 | 5.9 | 7.0 | 0.2 |

*^a^* *N*: coordination numbers; *^b^* *R*: bond distance; *^c^* *σ*^2^: Debye-Waller factors; *^d^* Δ*E*_0_: the inner potential correction. *R* factor: goodness of fit. *Ѕ*_0_^2^ were set as 0.83/0.88 for Ni-O/Ni-Ni, which were obtained from the experimental EXAFS fit of reference Ni foil/NiO by fixing CN as the known crystallographic value and was fixed to all the samples.

**Table S2.** Comparison of the HER performance for N-Ni©NC with recently reported Ni-based catalysts.

| **Catalyst** | **Overpotential**  **(10 mA cm^-2^)** | **Electrolyte** | **Reference** |
| --- | --- | --- | --- |
| **N-Ni**©**NC** | **27 mV** | **1.0 M KOH** | ***This work*** |
| CPF-Fe/Ni | 42 mV | 1.0 M KOH | *Nat. Commun. 14 (2023) 1792* |
| WN-Ni@N,P-CNT | 70 mV | 1.0 M KOH | *Appl. Catal. B: Environ. 298 (2022) 120511* |
| Ni-Mo_2_C/CF | 81 mV | 1.0 M KOH | *Chem. Eng. J. 427 (2022) 131712* |
| NiIr_SAA_-NiFe-LDH | 28.5 mV | 1.0 M KOH | *ACS Catal. 13 (2023) 11195–11203* |
| Ni@NCW-2.0 | 158 mV | 0.5 M H_2_SO_4_ | *Adv. Mater. 36(2024) 2304917* |
| NCNT-NP@NF | 96.1 mV | 1.0 M KOH | *Chem. Eng. J. 413 (2021) 127531* |
| Ni_SA_-O/Mo_2_C | 133 mV | 1.0 M KOH | *Nat. Commun. 15 (2024) 1342* |
| Co/Ni@GC/NCNTs/CNFs | 150 mV | 1.0 M KOH | *Compos. Part. B: Eng. 231 (2022) 109573* |
| CNTs/MoC/CoNiMo | 51 mV | 1.0 M KOH | *Appl. Catal. B: Environ. 343 (2024) 123470* |
| Co_3_O_4_@NiP_x_ | 99 mV | 1.0 M KOH | *Chem. Eng. J. 485 (2024) 149903* |
| CoNi-Ti_3_C_2_T_x_ | 31 mV | 1.0 M KOH | *ACS Nano 18 (2024) 4256–4268* |
| Ni_2_P-NiSe_2_/MoO_x_/NF | 23 mV | 1.0 M KOH | *Appl. Catal. B: Environ. 347 (2024) 123817* |
| V-Ni_3_FeN/Ni@N-GTs | 66 mV | 1.0 M KOH | *J. Mater. Chem. A 10 (2022) 18877-18888* |
| Pd-e-NiCo-PBA-C | 147 mV | 1.0 M KOH | *Adv. Funct. Mater. 31 (2021) 2008989* |

**Table S3.** Comparison of the OER performance for N-Ni©NC with recently reported Ni-based catalysts.

| **Catalyst** | **Overpotential**  **(10 mA cm^-2^)** | **Electrolyte** | **Ref.** |
| --- | --- | --- | --- |
| **N-Ni**©**NC** | **206 mV** | **1.0 M KOH** | ***This work*** |
| CPF-Fe/Ni | 194 mV | 1.0 M KOH | *Nat. Commun. 14 (2023) 1792* |
| WN-Ni@N,P-CNT | 268 mV | 1.0 M KOH | *Appl. Catal. B: Environ. 298 (2022) 120511* |
| FeNi-Mo_2_C/CF | 228 mV | 1.0 M KOH | *Chem. Eng. J. 427 (2022) 131712* |
| a-Ni/CeO_2_@NC | 286 mV | 1.0 M KOH | *Sci. Adv. 9 (2023) 1320* |
| Ni/CeO_2_@N-CNFs | 230 mV | 1.0 M KOH | *Angew. Chem. Int. Ed. 62 (2023) e202306333* |
| NCNT-NP@NF | 240 mV | 1.0 M KOH | *Chem. Eng. J. 413 (2021)* *127531* |
| Ni_SA_-O/Mo_2_C | 299 mV | 1.0 M KOH | *Nat. Commun. 15 (2024) 1342* |
| Co/Ni@GC/NCNTs/CNFs | 395 mV | 1.0 M KOH | *Compos. Part. B: Eng. 231 (2022) 109573* |
| CeO_2_/NiCo_2_S_4_ | 146 mV | 1.0 M KOH | *Appl. Catal. B: Environ. 344 (2024) 123659* |
| CoNi-Ti_3_C_2_T_x_ | 241 mV | 1.0 M KOH | *ACS Nano 18 (2024) 4256–4268* |
| Ni_2_P-NiSe_2_/MoO_x_/NF | 241 mV | 1.0 M KOH | *Appl. Catal. B: Environ. 347 (2024) 123817* |
| V-Ni_3_FeN/Ni@N-GTs | 252 mV | 1.0 M KOH | *J. Mater. Chem. A 10 (2022) 18877-18888* |
| Pd-e-NiCo-PBA-C | 309 mV | 1.0 M KOH | *Adv. Funct. Mater. 31 (2021) 2008989* |
| Ni/Fe_3_O_4_/IF | 210 mV | 1.0 M KOH | *J. Energy Chem. 73 (2022)* *330-338* |

**Table S4.** Concentrations of residual Ni^2+^ in electrolyte after HER and OER stability tests.

|  | Catalyst | Ni concentration (mmol L^-1^) |
| --- | --- | --- |
| HER | N-Ni©NC | 2×10^-4^ |
| OER |  | 3×10^-3^ |

**Table S5.** TOF values of N-Ni©NC and Ni©C in HER and OER.

|  | Overpotential | TOF (s^-1^) | |
| --- | --- | --- | --- |
|  |  | N-Ni©NC | Ni©C |
| HER | 100 mV | 1.844 | 0.234 |
| OER | 250 mV | 0.362 | 0.080 |

**References**

[1] P. E. Blöchl, *Phys. Rev. B* **1994**, *50*, 17953-17979.

[2] G. Kresse, J. Furthmüller, *Comp. Mater. Sci.* **1996**, *6*, 15-50.

[3] G. Kresse, J. Furthmüller, *Phys. Rev. B* **1996**, *54*, 11169-11186.

[4] J. P. Perdew, K. Burke, M. Ernzerhof, *Phys. Rev. Lett.* **1996**, *77*, 3865-3868.

[5] G. Kresse, D. Joubert, *Phys. Rev. B* **1999**, *59*, 1758-1775.
